# The HIF‐1α Pathway Regulates Satellite Cell Fate During Aging Through Histone Lactylation

**DOI:** 10.1111/acel.70411

**Published:** 2026-02-13

**Authors:** Marco Piccoli, Lorenzo Mornatti, Ivana Lavota, Monica Risuglia, Pasquale Creo, Elena Vizzino, Paola Rota, Adriana Tarantino, Laura Mangiavini, Giuseppe Ciconte, Paola Signorelli, Giuseppe Maria Peretti, Simone Cenci, Carlo Pappone, Luigi Anastasia, Federica Cirillo

**Affiliations:** ^1^ Laboratory of Stem Cells for Tissue Engineering IRCCS Policlinico San Donato Milan Italy; ^2^ University Vita‐Salute San Raffaele Milan Italy; ^3^ Institute for Molecular and Translational Cardiology (IMTC) IRCCS Policlinico San Donato Milan Italy; ^4^ Department of Biomedical, Surgical and Dental Sciences University of Milan Milan Italy; ^5^ Department of Biomedical Sciences for Health University of Milan Milan Italy; ^6^ IRCCS Istituto Ortopedico Galeazzi–Sant'Ambrogio Milan Italy; ^7^ Arrhythmology Department, IRCCS Policlinico San Donato Milan Italy; ^8^ “Aldo Ravelli” Research Centre, Department of Health Sciences University of Milan Milan Italy; ^9^ Biochemistry Laboratory, IRCCS Policlinico San Donato Milan Italy; ^10^ IRCCS Ospedale San Raffaele Milan Italy

**Keywords:** aging, FG‐4592, hypoxia‐inducible factor‐1α, lactylation, quiescence, satellite cells

## Abstract

Aging‐associated sarcopenia is driven in part by the progressive loss of type II glycolytic fibers and the functional decline of their resident stem cells, the satellite cells (SCs). We show here that these defects result from attenuation of the hypoxia‐inducible factor‐1α (HIF‐1α) signaling pathway and can be reversed by pharmacological HIF‐1α reactivation. In the tibialis anterior muscle of 18‐month‐old C57BL/6J mice, HIF‐1α protein abundance decreased by ≈46% and canonical targets (*Vegf*a, *Egln1*) were downregulated in freshly isolated SCs. Treatment of aged SCs with the prolyl hydroxylase inhibitor roxadustat (FG‐4592) for 48 h restored HIF signaling, upregulated glycolytic enzymes (HK2, GAPDH, ALDO) and the lactate transporter MCT4, and increased intracellular lactate by 1.9‐fold. Increased lactate enhanced global histone lactylation, an epigenetic mark that decreased with age. The effect was attenuated by the LDHA inhibitor oxamate, establishing a link between HIF‐driven metabolism and chromatin remodeling. HIF‐1α activation slowed old SC proliferation (S phase −60%), but decreased the senescence marker p16Ink4a (−54%) and increased the stem cell factor *Pax7* (+1.8‐fold), indicating a shift from senescence to a quiescent, regenerative state. When differentiation was induced without drugs, pretreated aged SCs formed hypertrophic myotubes (differentiation index +1.7), exhibited higher ATP content (+1.54‐fold), and activated the IGF‐1/PI3K–Akt–mTOR pathway, leading to an increase in tropomyosin (*Tpm1*) in fast fibers. These results suggest a HIF‐1α‐lactate‐lactylation axis that rejuvenates aged satellite cells and enhances myogenic performance, providing a mechanistic rationale for repurposing roxadustat to alleviate sarcopenia.

## Graphical abstract

FG‐4592–induced activation of HIF‐1α enhances anaerobic glycolysis and promotes lactate‐driven PAX7 lactylation, thereby boosting satellite cell activity and counteracting age‐related muscle decline.

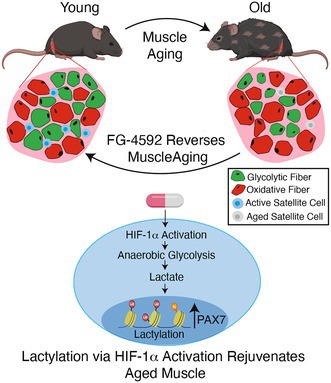

## 1 Introduction

Sarcopenia, originally defined in 2010 as low muscle mass with impaired physical function (Cruz‐Jentoft et al. 2010), was updated in 2019 to emphasize low muscle strength as the primary diagnostic criterion (Cruz‐Jentoft et al. 2019). The syndrome now represents a major public health burden, as it is associated with disability and functional limitations (Janssen et al. 2002), increased risk of falls (Scott et al. 2014), prolonged hospitalization (Gariballa and Alessa 2013), and escalating healthcare costs. Among non‐pharmacologic countermeasures, exercise training remains the most effective, consistently improving strength, muscle mass, and sarcopenia prevalence in older adults (Granacher et al. 2013; Petrella et al. 2007; Seo and Lee 2022; Steffl et al. 2017). To support this, the World Health Organization recommends ≥ 150 min/wk. of moderate‐intensity or ≥ 75 min/week of vigorous‐intensity exercise for older people (Bull et al. 2020; Okely et al. 2021). Physical activity slows age‐related muscle loss and partially preserves type II (fast‐twitch, glycolytic) fibers (Murgia et al. 2017; Nilwik et al. 2013).

Skeletal muscle comprises one slow (type I) and three fast (IIA, IIX, IIB) fiber classes arranged along a continuum from oxidative to glycolytic fibers (Schiaffino and Reggiani 2011). With increasing age, glycolytic fibers are preferentially degraded, which is accompanied by a quantitative and qualitative decline in resident stem cells, the satellite cells (SCs), impairing regeneration and accelerating atrophy (Verdijk et al. 2012; Verdijk et al. 2007). Oxygen tension is a crucial extrinsic regulator of SC behavior (Simon and Keith 2008; Yang et al. 2017). Cellular adaptation to hypoxia is controlled by hypoxia‐inducible factors (HIFs) (Huang et al. 1996), heterodimers consisting of an O₂‐labile α‐subunit and a constitutive β‐subunit (Huang et al. 1996; Wang and Semenza 1995). Under normoxia, enzymes of the prolyl hydroxylase domain (PHDs) hydroxylate HIF‐α, making it accessible for ubiquitin‐mediated degradation; hypoxia or PHD inhibition stabilizes HIF‐α and enables the transcription of angiogenic and glycolytic genes (Huang et al. 1996). Of the α‐isoforms (HIF‐1α/‐2α/‐3α) (Drevytska et al. 2012; Simon and Keith 2008), HIF‐1α predominates in SCs, whereas HIF‐2α is localized in myofibers after injury (Yang et al. 2017). Conditional loss of both isoforms in SCs impairs regeneration (Yang et al. 2017), while pharmacological HIF‐1α activation increases myofiber size in vitro and in vivo (Cirillo et al. 2020, 2017). Importantly, HIF‐1α signaling decreases with human aging and correlates with sarcopenic status (Cirillo et al. 2022), suggesting impaired HIF activity as a cause of disease.

HIF‐1α is a master regulator of glycolytic enzymes, many of which are downregulated in aged muscle. Recent work shows that lactate, the end product of anaerobic glycolysis, also acts as a signaling agent: it donates lactyl groups to histone lysines, resulting in a new epigenetic modification that influences gene expression (Zhang et al. 2019). Histone lactylation supports stem cell self‐renewal and modulates macrophage polarization (Zhang et al. 2019). It is noteworthy that lactylation decreases in aged skeletal muscle and can be restored by increasing glycolytic flux (Meng et al. 2025), highlighting its potential importance in muscle aging. However, whether reduced glycolysis and lactylation in aging satellite cells causally contribute to their dysfunction is still unknown.

Here we test the hypothesis that pharmacological reactivation of HIF‐1α restores glycolytic metabolism and histone lactylation in aged satellite cells, thereby preserving their stem cell capacity and enhancing their myogenic performance.

## 2 Results

### 2.1 Age‐Related Suppression of the HIF‐1α Pathway in Skeletal Muscle and Satellite Cells

**FIGURE 1:**
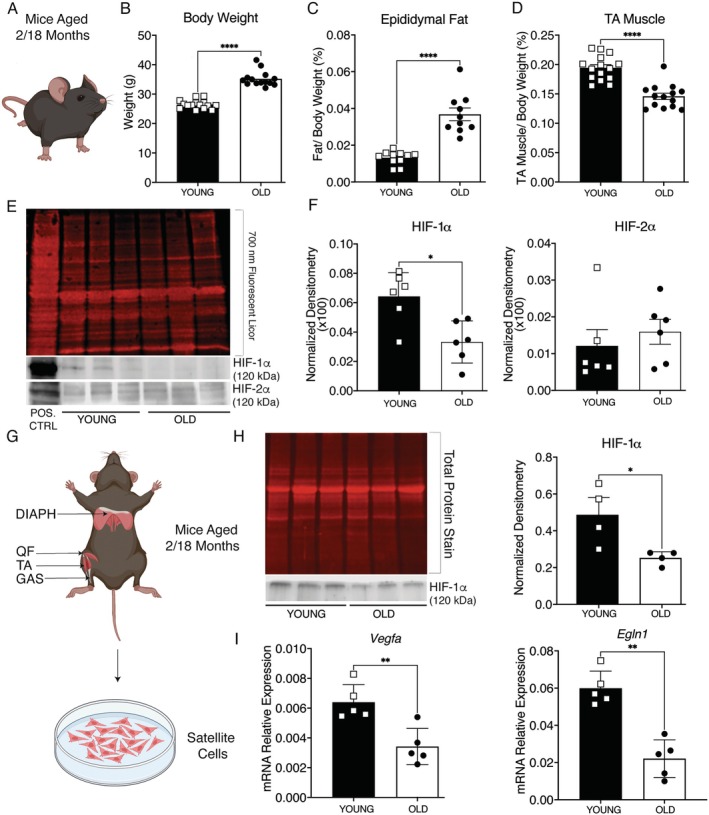
Age‐related decline of HIF‐1α in muscle tissue. (A) Schematic representation of mouse model. (B–D) Evaluation of body weight, epididymal fat, and tibialis anterior (TA) muscle mass in young (2‐month) vs. old (18‐month) male C57BL/6J mice. (E) Representative Western blot for HIF‐1α and HIF‐2α proteins in TA muscle lysates from young and old mice (with FG‐4592‐treated C2C12 cell lysate as a positive control for band identification). (F) Quantification of HIF‐1α and HIF‐2α protein levels in TA, normalized to total protein loading (Revert 700 stain). (G) Schematic representation of Satellite Cell isolation. (H) Representative Western blot and quantification of HIF‐1α protein in freshly isolated SCs from young and old mice. Results were normalized to total protein loading (Revert 700 stain). (I) Gene expression of HIF‐1α target genes: (i) *Vegfa* and (ii) *Egln1*. The data represent mean ± SEM. Statistical significance was determined by Mann–Whitney test. *p < 0.05, **p < 0.01, ****p < 0.0001.

To confirm that sarcopenia in our colony is associated with a decrease in HIF‐1α signaling, we first established a phenotypic baseline in male C57BL/6J mice (Figure 1A). Eighteen‐month‐old animals exhibited a 1.33‐fold increase in body weight, a 2.77‐fold increase in epididymal fat, and a 27% loss of tibialis anterior (TA) mass compared to 2‐month‐old controls (Figure 1B–D). Immunoblotting of TA extracts revealed that HIF‐1α protein abundance decreased by 46% with age, whereas HIF‐2α levels remained unchanged (Figure 1E,F). As demonstrated in our previous work, the specificity of the HIF‐1α and HIF‐2α antibodies was validated in C2C12 cells treated with FG‐4592, a clinically approved prolyl hydroxylase inhibitor that blocks PHD2 activity and thereby promotes HIF accumulation (Cirillo et al. 2020) (Figure S1). Accordingly, lysates from FG‐4592–treated C2C12 myoblasts were included as a positive control to facilitate band identification in muscle tissue.

Next, we investigated whether the reduced HIF‐1α in whole muscle reflected changes in the stem cell compartment. Satellite cells (SCs) were harvested from diaphragms, quadriceps, TA, and gastrocnemius muscles and subjected to negative selection for CD11, CD31, CD45, CD34, and positive selection for CD106 (Boscolo Sesillo et al. 2020; Pei 1999; Spada et al. 2016) (Figure 1G, Figure S2a). Flow cytometry confirmed that 81.7% of the sorted population was CD106^+^ (Figure S2a, panel ii), while each exclusion marker accounted for less than 6% of events (Figure S2a, panels iii‐vi).

To assess SC preparation quality, we analyzed canonical markers of SCs, skeletal muscle fibroblasts, adipocytes, and endothelial cells (Figure S2b). Pax7 was detected only in SCs, confirming successful enrichment (Figure S2b, panel i). Non‐myogenic contamination was minimal, as fibroblast markers (*Thy1*, *Pdgfra*, *Fabp4*) were reduced by 84%, 67%, and 96%, adipocyte markers (*Pparg*, *Plin1*) by 99%, and endothelial markers (*Pecam‐1*, *Vwf*) by 99% compared to their respective cell types (Figure S2b, panels ii–iv). These results confirm that SC preparations were highly enriched and consistent with the expected heterogeneity of primary muscle‐derived samples.

Label‐free LC–MS on freshly isolated SCs revealed an enrichment in metabolic pathways with a shift toward fatty acid β‐oxidation, oxidative phosphorylation, and lipolysis—metabolic signatures associated with myosteatosis and impaired regenerative capacity (Al Saedi et al. 2022; Tang et al. 2023) (Figure S3a–d, Table S1). Immunoblotting confirmed a 48% reduction in HIF‐1α protein levels in aged SCs (Figure 1H, panel i‐ii). Parallel qPCR showed that *Vegfa* and *Egln1* transcripts decreased by 46%, and 62%, respectively (Figure 1I, panels i‐ii). Overall, skeletal muscle tissue and SCs show a coordinated downregulation of HIF‐1α signaling with increasing age, providing a mechanistic basis for the sarcopenic phenotype observed in Figure 1A.

### 2.2 Pharmacologic Re‐Activation of HIF‐1α Reinstates Glycolytic Metabolism in Aged Satellite Cells

Since the age‐related metabolic drift of SCs is associated with the loss of HIF‐1α, we tested whether acute stabilization of the factor can reset their metabolic program. Young and old SCs were exposed to FG‐4592 at a concentration of 50 μM for 48 h. qPCR confirmed activation of the metabolic pathway, with *Vegfa* and *Egln1* increasing 3.17‐ and 6.97‐fold in young cells and 4.41‐ and 9.95‐fold in old cells, respectively (Figure S4a, panels i‐ii).

**FIGURE 2:**
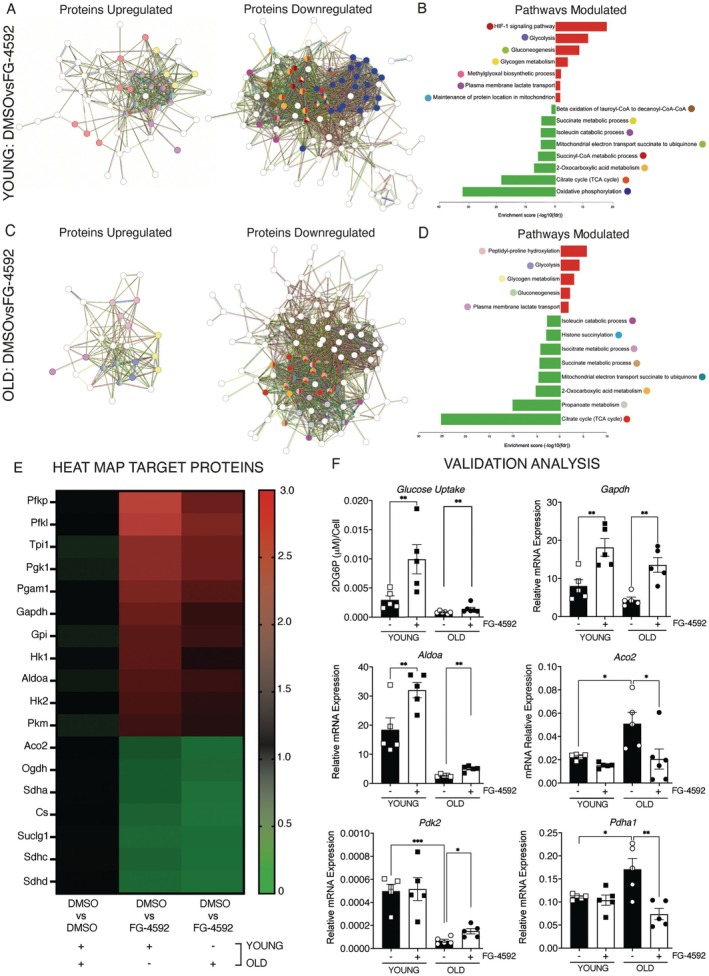
HIF‐1α activation reprograms metabolism from oxidative to glycolytic in satellite cells. (A, C) Protein–protein interaction network modulated by FG‐4592 treatment compared to control in young (A) and old (C) SCs. (B, D) GO analysis of functional enrichment and pathway database (KEGG, Reactome, Wikipathways) by SRPlot in young (B) and old (D) SCs. (E) Heatmap of 18 proteins significantly modulated by FG‐4592 treatment with a *p*‐value ≤ 0.05 and a log_2_FC ≥ 0.5. The color in each tile represents the scaled abundance value. The first column represents the modulation in SCs control during aging, while the second and the third show the modulation induced by FG‐4592 in young and old SCs, respectively. (F) Validation of proteomic findings was performed by measuring (i) glucose levels and (ii–vi) mRNA expression of key metabolic enzymes. Among these, *Gapdh* (ii), *Aldoa* (iii), and *Aco2* (vi) were identified in the proteomic dataset, while *Pdk2* (iv) and *Pdha1* (v) were additionally assessed due to their regulatory role in metabolic reprogramming. Data are presented as mean ± SEM. Statistical significance was determined using one‐way ANOVA. *p < 0.05, **p < 0.01, ***p < 0.001.

Proteomic profiling captured the global cellular response (Figure S4b,c). KEGG and Reactome analysis assigned the upregulated enzymes to glycolysis, glycogen degradation, and lactate export across the plasma membrane, whereas the downregulated enzymes were associated with the tricarboxylic acid cycle, the electron transport chain, and the branched‐chain amino acids catabolism (Figure 2A–D, Table S1). Analysis of individual enzymes confirmed the metabolic switch: Hk1, Hk2, Gapdh, Aldoa, and Pkm increased two‐ to fivefold, while Aco2, Sdha, and citrate synthase decreased significantly (Figure 2E).

To test whether these changes had functional consequences, we measured glucose uptake and sentinel transcript expression. Glucose uptake increased 3.41‐fold in young SCs and 1.96‐fold in old SCs (Figure 2F, panel i), and the mRNAs of *Gapdh* and *Aldoa* increased concordantly (Figure 2F, panels ii and iii). *Aco2*, which was increased 2.27‐fold with age, decreased 60% after FG‐4592 treatment (Figure 2F, panel iv). Next, we examined the Pdha1‐Pdk2 axis, which channels pyruvate into the mitochondria. *Pdk2*, which was suppressed 87% by aging, increased 2.25‐fold again, whereas *Pdha1* mRNA, which was increased 1.54‐fold in untreated old cells, decreased 57% after treatment (Figure 2F, panels v and vi).

These results demonstrate that a transient pharmacologic increase in HIF‐1α is sufficient to restore a glycolytic, youthful metabolic profile in aged SCs.

### 2.3 Lactate Accumulation and Recovery of Histone Lactylation in Aged Satellite Cells

**FIGURE 3:**
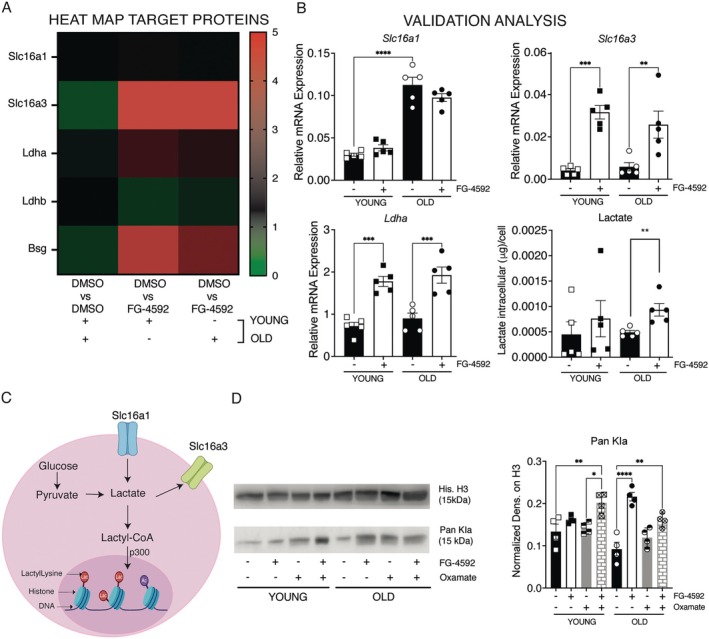
HIF‐1α activation increases lactate production and restores histone lactylation in aged SCs. (A) Heatmap of proteins modulated by FG‐4592 treatment and involved in the lactate pathway in young and old SCs. (B) Validation analysis of LC–MS results by measuring the gene expression analysis of (i) *Slc16a1*, (ii) *Slc16a3*, and (iii) *Ldha* by Real‐Time PCR and (iv) quantifying the intracellular lactate accumulation in young and old SCs. (C) Schematic representation of histone lactylation induced by the transfer of lactyl groups to the lysine residues on the chromatin histone by acetyltransferase p300. (D) Evaluation of histone lactylation levels (Pan Kla) in young and old SCs after FG‐4592 treatment with or without oxamate, an inhibitor of Ldha. The graph on the right shows the quantification analysis of the western blot normalized on histone H3. Data represent mean values ± SEM. Statistical significance was determined by one‐way ANOVA. *p < 0.05, **p < 0.01, ***p < 0.001.

Next, we investigated whether metabolic rewiring leads to increased lactate production and consequent histone lactylation, a modification that plays a role in stem cell maintenance (Zhang et al. 2019). Proteomics analysis revealed robust induction of *Ldha* and the lactate exporter *Slc16a3* (MCT4), whereas the importer *Slc16a1 (*MCT1) or LDHB remained unchanged (Figure 3A). qPCR confirmed a 7.56‐fold induction of Slc16a3 and a 2.3‐fold induction of *Ldha* in young cells, and a 4.31‐ and 2.1‐fold increase in old cells, respectively (Figure 3B, panels i‐iii). Intracellular lactate increased 1.9‐fold in old SCs (Figure 3B, panel iv), leading to potential involvement of histone lactylation (Figure 3C).

To investigate the relation between SCs aging and lactylation, SCs were treated with 10 mM oxamate, a chemical inhibitor of LDHA (Hashimoto et al. 2025). Proteomic analysis showed that oxamate alone did not substantially alter the profiles of young or old SCs compared to DMSO controls (data not shown). SCs co‐treated with oxamate and FG‐4592 exhibited proteomic and metabolic profiles largely overlapping with FG‐4592‐only treatment, indicating that oxamate does not fully counteract FG‐4592–induced transcriptional activation (Figure S5a–c, Table S1). This is likely because FG‐4592 upregulates *Ldha* expression more strongly than oxamate inhibits it, allowing SCs to maintain glycolytic and metabolic shifts triggered by HIF‐1α stabilization, as shown in proteomic analysis (Figure S5a–c). Upregulated pathways with co‐treatment included glycolysis, HIF signaling, and gluconeogenesis, while downregulated pathways involved the Krebs cycle and oxidative phosphorylation, mirroring FG‐4592‐only effects. (Figure S5c). Immunoblotting with a pan‐lactyl‐lysine antibody revealed lower baseline histone lactylation in old SCs compared to young SCs, though this difference was not statistically significant (Figure 3D, panels i and ii). Notably, FG‐4592 increased the signal in old cells by 2.37‐fold but did not alter young cells, indicating a ceiling effect (Figure 3D). Consistent with mass spectrometry analysis, oxamate alone did not alter lactylation levels compared with DMSO‐treated SCs in young and old mice (Figure 3D, panels i and ii). Interestingly, SCs co‐treated with FG‐4592 and oxamate displayed a 1.5‐ and 1.75‐fold increase in lactylation, indicating that lactate, rather than an off‐target effect of FG‐4592, is responsible for the observed chromatin modification (Figure 3D, panels i and ii).

### 2.4 Lactylation‐Linked Reprogramming of the Cell Cycle From Senescence to Quiescence

**FIGURE 4:**
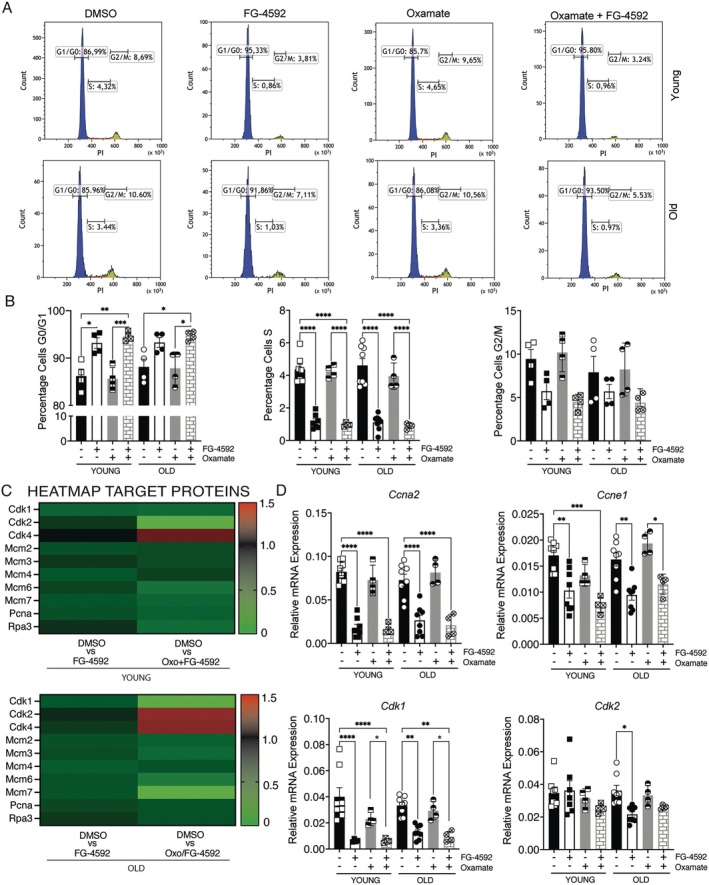
Transient HIF‐1α stabilization induces a cell cycle arrest resembling quiescence. (A) Cell cycle profile of controlled and treated SCs isolated from young and old mice. The percentage of cells in the G0‐G1 phase is shown in blue, the percentage of cells in the S phase in red, and the percentage of cells in the G2 phase in green. (B) Effects of FG‐4592 treatment on the accumulation of SCs in the G1, S, G2/M phase of the cell cycle compared to controls. (C) Heatmaps of cell cycle proteins modulated by treatment in young and old SCs. (D) Validation of LC–MS results by measuring gene expression of (i) *Ccna2*, (ii) *Ccne1*, (iii) *Cdk1*, and (iv) *Cdk2* by Real‐Time PCR. Data represent mean ± SEM. Statistical significance was determined by one‐way ANOVA. *p < 0.05, **p < 0.01, ***p < 0.001.

To assess whether increased lactylation affects cell‐cycle progression, satellite cells (SCs) were synchronized in serum‐free medium for 24 h, treated with FG‐4592 for 48 h, and stained with propidium iodide. Flow‐cytometry profiles are shown in Figure 4A, with gating in Figure 4B. Aging reduced S‐phase fraction by ~15%, consistent with senescence‐associated slowing (Figure 4B, panel ii) (Zhang et al. 2022). FG‐4592 further decreased S‐phase occupancy by 65% in young and 60% in aged SCs, increasing G1/G0 arrest (Figure 4B, panels i–iii). Co‐treatment with oxamate and FG‐4592 caused ~79% S‐phase reduction in both groups, whereas oxamate alone had no effect (Figure 4B, panel iii). These data were confirmed by proteomics analysis, which showed that each cyclin–CDK pair required for G1/S progression decreased after treatment, most notably the cyclin E‐CDK2 and cyclin A2‐CDK1 modules (Figure 4C). qPCR confirmed these changes. In young SCs, FG‐4592 treatment significantly reduced the expression of *Ccna2* (77%), *Ccne1* (42%), and *Cdk1* (85%), while *Cdk2* levels were not significantly altered (Figure 4D, panels i‐iv). Similar reductions were observed in old SCs. In particular, *Ccna2* decreased by 67%, *Ccne1* by 42%, *Cdk1* by 64%, and *Cdk2* by 58% (Figure 4D, panels i‐iv). qPCR analysis confirmed that oxamate alone did not affect expression of cyclin genes, whereas co‐treatment strongly reduced these targets (Figure 4D, panels i‐iv). In young SCs, *Ccna2*, *Ccne1*, and *Cdk1* decreased by 81%, 56%, and 85%; in old SCs, *Ccna2*, *Ccne1*, and *Cdk1* dropped by 73%, 30%, and 72%, while *Cdk2* remained unchanged (Figure 4D, panels i–iv).

**FIGURE 5:**
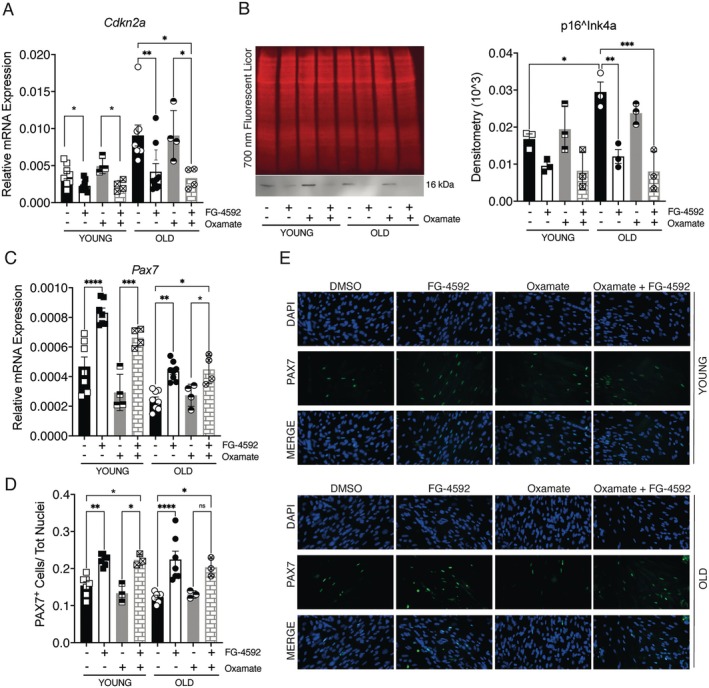
HIF‐1α activation reduces senescence and enhances the stem cell marker Pax7 in SCs. (A, B) Effects of FG‐4592 treatment on *Cdkn2a* gene expression by Real‐Time PCR and intracellular accumulation of p16^Ink4a^ by western blot in young and old SCs. Total protein staining was used to normalize the quantification. (C) Effects of FG‐4592 treatment on *Pax7* gene expression. (D‐E) Validation of Pax7 results by analyzing protein expression by immunofluorescence. Control and treated SCs were stained with an antibody that recognizes Pax7 (green), and nuclei were stained with 4′,6‐diamidino‐2‐phenylindole (DAPI). Quantification of Pax7‐positive cells on the total number of nuclei in young and old SCs treated with FG‐4592 compared to control. Data represent mean ± SEM. Statistical significance was determined by one‐way ANOVA. *p < 0.05, **p < 0.01, ***p < 0.001.

To distinguish quiescence from senescence, *Cdkn2a* and *Pax7* were examined. Aging increased *Cdkn2a* transcripts 2.43‐fold and p16^Ink4a^ protein 1.76‐fold (Figure 5A). FG‐4592 reduced *Cdkn2a* and p16^Ink4^a by ~40% in young SCs (not significant) and by 67% and 54% in old SCs, restoring youthful levels. Oxamate + FG‐4592 mirrored this pattern, with *Cdkn2a* decreased 54% in young and 64% in old SCs; p16^Ink4a^ reduction was significant only in old SCs (64%) (Figure 5A,B). In parallel, *Pax7* expression also improved following HIF‐1α activation. *Pax7* mRNA levels increased 1.80‐fold in young SCs and 1.79‐fold in old SCs after FG‐4592 treatment (Figure 5C). Co‐treatment with oxamate and FG‐4592 produced a similar trend, upregulating *Pax7* transcripts by 1.43‐fold in young SCs and 1.83‐fold in old SCs (Figure 5C). Immunofluorescence confirmed that the proportion of Pax7^+^ nuclei increased 1.46‐fold in young cultures and 1.83‐fold in old cultures after FG‐4592 exposure (Figure 5D,E). Co‐treated SCs showed a response comparable to that induced by FG‐4592 alone, with a 1.46‐fold and 1.66‐fold increase in Pax7^+^ nuclei in young and old SCs, respectively (Figure 5D,E).

These results indicate HIF‐1α–driven lactylation shifts aged SCs from senescence toward quiescence, reducing p16^Ink4a^ and restoring Pax7.

### 2.5 Sustained Glycolytic Reprogramming Enhances Myogenic Differentiation After Transient HIF‐1α Activation

**FIGURE 6:**
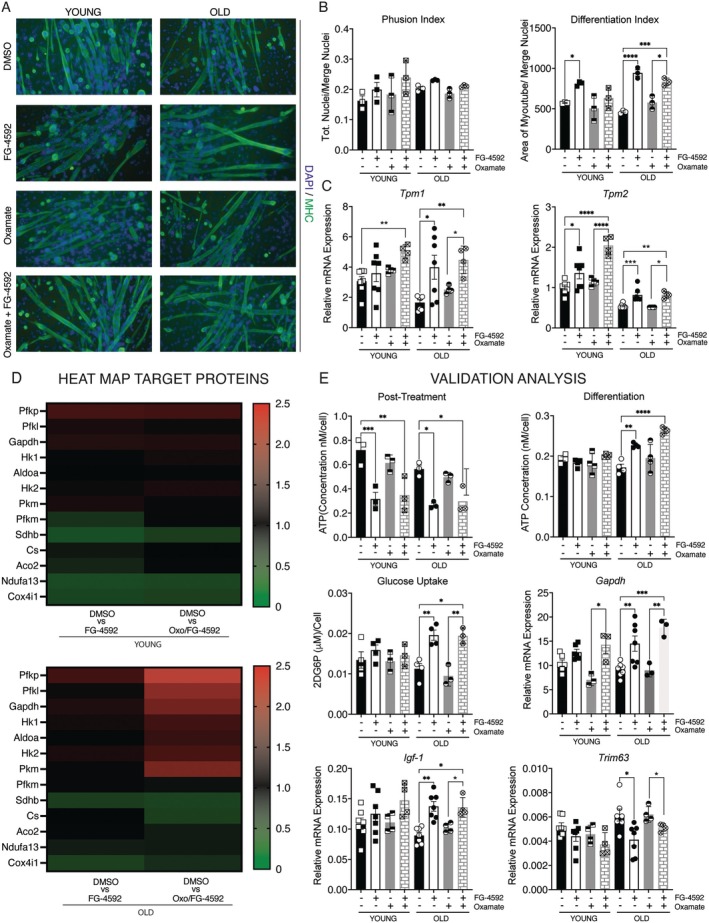
A short HIF‐1α‐activation pulse enhances subsequent myotube hypertrophy in vitro. (A) Immunofluorescence of MHC (green) on young and old myotubes obtained from SCs control and treated with FG‐4592. (B) Two parameters shown in the graphs below were considered: The fusion index, measured as the number of merged nuclei on the total number of nuclei, and the differentiation index, measured as the area of myotubes on the merged nuclei. (C) Effects of FG‐4592 treatment on the *Tpm1* and *Tpm2* gene expression in young and old myotubes. (D) Heatmap of 13 proteins significantly modulated by FG‐4592 and co‐treatment during skeletal muscle differentiation with a *p*‐value ≤ 0.01 and a log_2_FC ≥ 0.5. Results were compared with DMSO‐control myotubes. The color in each tile represents the scaled abundance value. (E) Validation analysis of proteomic data measuring: ATP concentration in young and old SCs following treatment (panel i) and after 4 days of differentiation (panel ii), glucose uptake (panel iii), Gapdh (panel iv), *Igf‐1* (panel v), and *Trim63* (panel vi) gene expression. Data represent mean ± SEM. Statistical significance was determined by one‐way ANOVA. *p < 0.05, **p < 0.01.

To test whether a short HIF‐1α pulse confers lasting benefits, SCs were pretreated for 48 h with FG‐4592, oxamate, or both drugs, washed, and cultured in drug‐free differentiation medium for 4 days. Myosin heavy chain immunostaining showed that the fusion index was unchanged, but the differentiation index, calculated as myotube area per fused nucleus, increased 1.6‐fold in young cultures and 2‐fold in old cultures, indicating larger fibers (Figure 6A,B). Oxamate alone did not affect SC differentiation, whereas co‐treatment increased the differentiation index 1.8‐fold in old myotubes (Figure 6A,B). Tropomyosin‐1 (*Tpm1*) and Tropomyosin‐2 (*Tpm2*) expression confirmed enhanced differentiation. Aging reduced *Tpm1* transcript by 46%, but FG‐4592 restored it 2.41‐fold in old myotubes, comparable to young controls; co‐treatment induced a 2.7‐fold increase (Figure 6C). In young myotubes, FG‐4592 had little effect, while co‐treatment increased *Tpm1* 1.63‐fold. *Tpm2* showed a similar trend: decreased with aging (−54%), upregulated by FG‐4592 in young and old (1.35‐ and 1.5‐fold), and further maintained during co‐treatment (2‐ and 1.5‐fold) (Figure 6C). Overall, FG‐4592 restored *Tpm1* and *Tpm2* in old myotubes to youthful levels.

LC–MS of myotube lysates showed that DMSO‐cultures exhibited age‐related depression of the hypoxia response, contractile machinery, and mitochondrial metabolism (Figure S6c,d, Table S2). In contrast, myotubes derived from FG‐4592‐primed SCs maintained high expression of HIF‐responsive glycolytic enzymes and low expression of components of oxidative phosphorylation (Figure 6D, Figure S7a–f, Table S2). The glycolytic pathway also remained upregulated in myotubes derived from co‐treatment (Figure 6D, Figure S8a–c, Table S2). Old myotubes also showed enhanced IG‐1 and PI3K–Akt–mTOR signaling, both drivers of hypertrophy (Musaro et al. 2001; Schiaffino and Mammucari 2011; Stitt et al. 2004) (Figure S7d, Table S2).

To confirm that metabolic pathway modulation persists during differentiation, we assessed changes in selected metabolic proteins (Figure 6E). In particular, ATP levels, which had declined during the resting phase in both FG‐4592‐treated (−57% and −54% in young and old myotubes) and co‐treated cells (−52% and −49% in young and old myotubes), recovered during differentiation (Figure 6E, panels i‐ii). Young myotubes showed no significant change, while old myotubes increased ATP 1.34‐ and 1.53‐fold after FG‐4592 or co‐treatment (Figure 6E, panels i–ii). Along this line, glucose uptake and *Gapdh* mRNA showed the most pronounced alterations in old myotubes. Specifically, glucose uptake increased 1.72‐fold in old myotubes from both treatments (Figure 6E, panel iii). This pattern was mirrored by *Gapdh* expression levels, which increased 1.58‐fold and 1.95‐fold in old myotubes derived from FG‐4592 alone or co‐treatment, respectively (Figure 6E, panel iv).

The anti‐atrophy effect of FG‐4592 was further supported by *Igf‐1* levels, which increased 1.54‐fold and 1.51‐fold in old myotubes following FG‐4592 or co‐treatment, respectively (Figure 6E, panel v). Consistently, the expression of *Trim63*—an established marker of atrophy—showed a downward trend (Figure 6E, panel vi) (Bodine and Baehr 2014). Lower p16^Ink4a^ levels in parental SCs correlated with these differentiation gains. Thus, a short HIF‐1α stimulus installs metabolic and epigenetic memory that sustains glycolysis, activates growth‐promoting signals, and produces larger, functionally superior myotubes, even after drug withdrawal.

## 3 Discussion

Low oxygen tension is a defining feature of stem cell niches and is essential for preserving self‐renewal and regenerative competence (Scadden 2006). Although direct real‐time measurements of niche PO₂ in vivo remain challenging, estimates in human tissue range from 3% to 13% O₂ (Mas‐Bargues et al. 2019). Within this range, the hypoxia‐inducible factors HIF‐1α and HIF‐2α orchestrate transcriptional programs that enable stem cells to couple energy production to low oxygen availability while maintaining their pluripotency and developmental potential (Cui et al. 2021; Mathieu et al. 2014; Takubo et al. 2010). In skeletal muscle, microvascular pO₂ is naturally lower in predominantly glycolytic, fast‐twitch muscles such as the digitorum longus extensor than in the oxidative, slow‐twitch soleus (McDonough et al. 2005). This physiological gradient explains the higher HIF‐1α concentrations previously reported in fast muscles (Lunde et al. 2011), and underscores a central role of HIF‐1α in maintaining type II fiber morphology and metabolism. Conversely, HIF‐2α integrates oxidative cues by regulating Pgc‐1α and promoting slow fiber gene expression; muscle‐specific HIF‐2α deletion shifts fibers toward type IIb phenotype (Rasbach et al. 2010).

Our data extend these observations to aging, showing a coordinated decrease in HIF‐1α protein and its canonical target genes in the tibialis anterior and in freshly isolated SCs. Although Phd2 levels decrease with age, this does not necessarily stabilize HIF‐1α, because its stability is also influenced by oxygen availability, co‐factors (Fe^2+^, 2‐oxoglutarate, ascorbate), mitochondrial ROS, and NAD^+^ levels, all of which are altered in aged muscle stem cells (Yeo 2019; Zhang et al. 2025). Moreover, aging is associated with increased VHL‐mediated degradation and impaired HIF‐1α transcription, so the net effect is reduced HIF‐1α availability despite lower Phd2 protein (Joo et al. 2023).

The suppression of HIF‐1α is closely associated with the loss of type II fibers, which rely on glycolytic metabolism downstream of HIF‐1α. Previous studies show that the combined HIF‐1α and HIF‐2α deletion in SCs impairs regeneration after injury (Yang et al. 2017), which is in line with the reduction in SCs number described in biopsies from sarcopenic patients (Cirillo et al. 2022). Consistent with these findings, our proteomic analysis showed that aged SCs upregulate fatty acids β‐oxidation and oxidative phosphorylation at the expense of glycolysis, metabolic programs incompatible with rapid activation and proliferation. The notion that hypoxic signaling preserves the potential of satellite cells is also supported by previous work showing that brief exposure to 1% O₂ improves self‐renewal, grafting efficiency, and differentiation (Cirillo et al. 2022; Liu et al. 2012).

Pharmacological re‐stabilization of HIF‐1α with FG‐4592, a clinically approved PHD inhibitor, restored glycolysis in both young and aged satellite cells. Untargeted proteomics revealed a 2‐ to 5‐fold increase in all rate‐limiting glycolytic enzymes examined, accompanied by suppression of the tricarboxylic acid cycle and electron transport chain proteins. Functional assays confirmed a 3‐to 2‐fold increase in glucose uptake and a complete reversal of the age‐related Pdh–Pdk imbalance, suggesting that metabolic reprogramming was not limited to protein abundance but resulted in true flux changes. Consistently, FG‐4592 was recently shown to enhance glycolysis and insulin‐stimulated glucose uptake in human myotubes, highlighting the HIF pathway's role in muscle metabolism (Makinen et al. 2024).

An important advance of this study is the demonstration that glycolytic rescue led to a parallel increase in intracellular lactate and, crucially, restored histone lactylation in aged SCs. Lactate‐dependent lysine lactylation was first described as an immunometabolic signal in macrophages (D. Zhang et al. 2019) and has since emerged as an epigenetic mark that promotes embryonic stem cell self‐renewal and reprogramming of induced pluripotent stem cells (Dong et al. 2024; Guo et al. 2021; Hu et al. 2024). Recently, Meng et al. reported that skeletal muscle lactylation decreases with age and can be restored by increasing glycolytic flux (Meng et al. 2025). Our work complements and significantly extends these findings by identifying satellite cells as a key responding cell type, mechanistically linking the rescue to HIF‐1α activation, and demonstrating downstream functional improvements in regenerative assays. The concordance between our studies strengthens the potential of lactylation as a gerotherapeutic target.

The increased histone lactylation appears to mediate SCs fate, as it coincided with a reduction in cyclin–CDK expression which decreased in the S‐phase fraction, lowered p16^Ink4a levels, and increased in *Pax7* transcripts and Pax7‐positive nuclei. These signatures indicate a shift from irreversible senescence to reversible quiescence, preserving long‐term stem cell competence. Pharmacological inhibition of Ldha with oxamate did not alter chromatin and cell cycle effects as compared to DMSO‐controls, whereas when SCs were co‐treated with oxamate and FG‐4592 again exhibited the same alterations, indicating that the activation of HIF‐1α pathway counteracts the chemical activity of oxamate directly inducing the gene expression of *Ldha* and enhancing lactate production.

The benefits persisted even after discontinuation of the drug. SCs exposed to a 48‐h pulse of FG‐4592 produced myotubes that were almost twice as large, contained more ATP and expressed more tropomyosin‐1/2. Proteomic analysis of these myotubes showed sustained activation of glycolysis and PI3K–Akt–mTOR and suppression of oxidative phosphorylation. The upregulation of *Igf‐1* and the partial downregulation of *Trim63* further emphasized the shift toward anabolism. These molecular changes resulted in measurable functional benefits, and the fact that the differentiation index improved without changes in the fusion index suggests that hypertrophy was achieved by increasing the size of individual fibers and not simply by increasing fusion events. Such preservation of fusion capacity is particularly attractive because excessive fusion can deplete the satellite cell pool in vivo. Notably, genetic or pharmacological PHD inhibition has shown enhanced muscle repair in vivo, supporting our in vitro findings and the potential of HIF‐pathway targeting in sarcopenia (Settelmeier et al. 2020).

A limitation of the present study is that the mechanistic insights are derived primarily from in vitro SCs experiments. Although previous in vivo work in young animals supports the potential of FG‐4592 to enhance muscle regeneration, future studies in aged models are necessary to validate the translational relevance of the HIF‐1α–lactate–lactylation axis and the efficacy of intermittent pharmacological activation (Cirillo et al. 2020).

Overall, the current study combines metabolic, epigenetic, and cell cycle observations into a single cascade that begins with age‐dependent HIF‐1α loss and culminates in impaired regeneration. Pharmacological rescue of the pathway reverses each step, restores lactate‐dependent chromatin modifications, and directs SCs fate from senescence to quiescence, as demonstrated by the strong reduction in the expression levels of p16. This aligns with previous findings that geriatric satellite cells become dysfunctional due to p16^Ink4a^‐driven geroconversion, and that reducing p16^Ink4a^ can rejuvenate their function (Sousa‐Victor et al. 2014). Finally, this treatment promotes a sustained hypertrophic program in newly formed myotubes.

### 3.1 Conclusions

Our research identifies the HIF‐1α–lactate–histone lactylation axis as a critical and druggable pathway governing satellite cell aging. We demonstrate that short, clinically feasible pulses of the PHD inhibitor FG‐4592 effectively rejuvenate aged satellite cells by restoring their glycolytic metabolism, resetting their epigenetic landscape, and reviving their regenerative competence. Crucially, this transient intervention imprints a lasting pro‐hypertrophic memory that enhances subsequent myogenic differentiation. These results provide a compelling mechanistic rationale for investigating the intermittent administration of FG‐4592, both alone and in combination with resistance training, as a promising therapeutic strategy to combat sarcopenia and other muscle‐wasting conditions.

## 4 Methods

### 4.1 Animals

Animal experiments were performed according to the guidelines for animal protocols described in the Suppression Authorization No. 01/2023 approved by the San Raffaele Scientific Institute (Milan, Italy). Mice were purchased from Charles River Laboratories (Calco, Italy) and housed for 2 weeks in individual cages with a 12‐h light–dark cycle and ad libitum access to food and water. In accordance with the Council Directive of the European Communities of 24 November 1986 (86/609/EEC), every effort was made to minimize animal suffering and reduce the number of mice used. Skeletal muscle tissues, including tibialis anterior, soleus, gastrocnemius, extensor digitorum longus, quadriceps, and diaphragm, were collected from age‐matched male mice aged 2 months (*n* = 15) and 18 months (*n* = 14).

### 4.2 Satellite Cells (SCs) Isolation

SCs were isolated from the muscles of 2‐ and 18‐month old mice C57BL/6J (Charles River). Mice were sacrificed by cervical dislocation to harvest the tibialis anterior, gastrocnemius, soleus, extensor digitorum longus (EDL), quadriceps, and diaphragm muscles, which were transferred to DMEM HG supplemented with 10% (v/v) FBS, 1% glutamine, and 2% penicillin/streptomycin. The muscle tissue was minced and placed in a tube containing 2% collagenase type II (Gibco), dissolved in DMEM HG supplemented with 1% penicillin/streptomycin (Euroclone), and incubated at 37°C for 60 min with agitation. The resulting homogenate was centrifuged at 400×*g* for 10 min, and the supernatant was discarded. The pellet was resuspended in DMEM HG + 10% HS and mechanically dissociated using serological pipettes. After dissociation, the pellet was allowed to settle by gravity for 5 min. The supernatant was collected in another tube containing DMEM HG + 10% HS to inhibit collagenase II activity. The dissociation was repeated twice. The collected supernatant was filtered in a cell strainer (40 μm, Corning) to obtain single cells. The cell mixture was incubated in DMEM HG + 10% HS for 1 h to reduce cellular contamination. After pre‐plating, cells were centrifuged at 500×*g* for 10 min and seeded into plates coated with Collagen I, Rat Tail (Gibco). Pre‐plated cells were cultured in low‐glucose DMEM (1 g/L; Merck) supplemented with 10% FBS (Merck), 1% glutamine (Merck), and 1% penicillin/streptomycin (Euroclone), and were used as skeletal muscle fibroblasts to assess SC preparation contamination.

Isolated SCs were cultured in growth medium (GM) consisting of Dulbecco's Modified Eagle's Medium (DMEM, Merck) with high glucose concentration (HG, 4.5 g/L) supplemented with 20% fetal bovine serum (FBS, Merck), 10% horse serum (HS, Merck), 1% glutamine (Merck), 1% penicillin/streptomycin (Euroclone), and 1% Chicken Embryo Extract (CEE, MP Biomedicals). The differentiation medium (DM) consisted of DMEM‐HG with 5% horse serum (HS, Merck). For pharmacological activation of the HIF‐1α pathway, SCs were treated with 50 μM FG‐4592 (Aurogene), a PHDs inhibitor, for 48 h. For lactylation studies, SCs were treated with 10 mM Oxamate, an inhibitor of lactate dehydrogenase (Ldha), or cotreated with 50 μM FG‐4592 in the presence of 10 mM Oxamate. These cells were tested for mycoplasma contamination prior to the experiments and were found to be negative.

### 4.3 Sample Preparation and Protein Digestion for Liquid Chromatography—Mass Spectrometry (LC–MS) Analyses

Total protein extracts from tissue and cell samples were prepared using the EasyPep Mini MS Sample Prep Kit (Thermo Fisher Scientific) according to the manufacturer's instructions.

To isolate tissue proteins, 5 mg of tibialis anterior muscles were homogenized using the TissueLyser II (Qiagen) in the presence of 100 μL of lysis solution and 1 μL of Universal Nuclease. After homogenization, samples were incubated on ice for 30 min and centrifuged at 16,000×*g* at 4°C for 10 min. The supernatant was collected and quantified using the Pierce BCA Assay Kit (Thermo Scientific) with a NanoDrop instrument (Thermo Fisher Scientific). For digestion, 100 μg of protein was incubated with a trypsin/Lys‐C protease mixture for 3 h at 37°C.

For protein isolation from SCs, the cell pellet was resuspended in 100 μL of lysis solution, treated with 1 μL of Universal Nuclease, and centrifuged at 13,000×*g* for 10 min at 4°C. The supernatant was quantified using the Pierce BCA, and 60 μg of the proteins were digested as previously described for tissue samples. For both tissue and cell protein samples, digestion was stopped with Digestion Stop Solution. Finally, the samples were transferred to dry Peptide Clean‐up Columns, and the peptides were eluted by adding the specific elution solution, followed by centrifugation.

### 4.4 Liquid Chromatography—Mass Spectrometry (LC–MS)

The chromatography system used consisted of an Ultimate 3000 RSLCnano system (Dionex, Germering, Germany) coupled online with a Q‐Exactive Plus mass spectrometer (Thermo Fisher Scientific). The LC system was used to perform sample injection, sample concentration, and LC separation automatically. It included an autosampler (Ultimate 3000 WPS‐3000TPL RS, Dionex, Thermo Fisher Scientific), a 10‐port injection valve (Valco Instruments), a binary nanoUPLC pump (Ultimate 3000 NCP‐3200RS, Dionex, Thermo Fisher Scientific), and an HPLC sample loading pump (Ultimate 3000 NCS‐3500RS, Dionex, Thermo Fisher Scientific). A Q‐Exactive Plus Orbitrap MS (Thermo Fisher Scientific) equipped with an Easy Spray source (Thermo Fisher Scientific) was used to analyze the separated peptides. The ESI spray voltage was set to 1.5 kV. The ion transfer capillary was heated to 250°C to desolvate the droplets. The RF level of the S‐lens was set to 50. Data‐dependent mode was used to automatically trigger the precursor scan and MS/MS scans.

The settings for the full scan were: Resolution of 70,000, 100 ms maximum injection time, automatic gain control (AGC), target value 3 × 10^6^. The MS2 settings were: Resolution of 17,500, AGC target of 2 × 10^5^, isolation window ±1.2 Da, and dynamic exclusion of 35.0 s. The precursor ions were excluded from fragmentation if they had an unassigned charge state or charge states +1, +7, +8, and higher than +8. Data acquisition was controlled by Xcalibur 2.11 and Tune software 4.4 (Thermo Fisher Scientific). All analyses were performed in duplicate.

For the analysis, a solution of 100 ng/μL of purified peptides was prepared with the loading solvent (0.1% formic acid in water) and 2 μL (equivalent to 200 ng) were injected and loaded onto the trap column (C18 PepMap100, 5 μm, 100 Å, 0.3 × 5 mm) in a mobile water phase containing 0.1% formic acid at 20 μL/min. After 2 min of loading, the trap cartridge was switched in‐line to an Easy‐Spray analytical column (PepMap RSLC C18, 2 μm, 25 cm × 75 μm) at 300 nL/min. Chromatography was performed at 40°C with mobile phase A (0.1% formic acid in water) and mobile phase B (0.1% formic acid in 80:20 acetonitrile/water, v/v). The chromatographic separation protocol included a non‐linear gradient: 35 min (3%–30% B), 5 min (30%–40% B), 5 min (40%–80% B), 4 min (80% B hold). After lowering to 3% B in 1 min, the column was equilibrated with 3% B for 10 min.

The LC eluent was sprayed into the mass spectrometer using an Easy‐Spray source (Thermo Fisher Scientific). After a complete scan in the range of *m/z* 375.0 to *m/z* 1500.0, a high‐energy collision dissociation (HCD) MS^2^ (28% NCE) was performed from the 20 most intense peaks (Top20) from the complete scan.

### 4.5 Flow Cytometry Analysis

Flow cytometric analysis was performed with approximately 1 × 10^6^ SCs. Nonspecific binding sites were blocked with a blocking solution containing 50% 1× phosphate‐buffered saline (PBS) and 50% FBS for 30 min at room temperature, followed by two washes with wash solution. Cells were stained with fluorochrome‐conjugated rat anti‐mouse antibody in wash solution (5% FBS in PBS) for 10 min on ice. After staining, cells were washed twice with wash solution at 4°C. SCs were stained with the following antibodies: αCD11 FITC, αCD106 APC (BioLegend, San Diego, CA), αCD31 APC (Thermo Fisher, Eugene, OR), αCD34 PE, αCD45 FITC (eBioscience, San Diego, CA). An unstained sample served as a negative control. Samples were acquired with a CytoFlex flow cytometer (Beckman Coulter), and data were processed with Kaluza 2.1 software (Beckman Coulter).

### 4.6 Gene Expression by Real‐Time Quantitative PCR (qPCR)

RNA was extracted using the Relia Prep RNA Miniprep System (Promega) and transcribed using the iScript cDNA Synthesis Kit (BioRad) according to the manufacturer's instructions. Quantitative real‐time PCR (qPCR) was performed with 10 ng cDNA template, 0.2 μM primers, and GoTaq qPCR Master Mix (Promega) in 20 μL final volume using a StepOnePlus Real‐Time PCR System (Applied Biosystem). Relative quantification of target genes was performed in duplicate and calculated according to the equation 2^−ΔΔCt^ using RPL1 as the housekeeper gene (see Table S3).

### 4.7 Total Protein Extraction From Tissue Sample

Tibialis anterior muscles were homogenized in 1 mL of TRIzol reagent (Fisher Molecular Biology) using a TissueLyser II (Qiagen) and then centrifuged at 12,000×*g* for 10 min. To isolate the phenol‐ethanol phase containing proteins, 200 μL of chloroform was added to the homogenate and then centrifuged. The proteins were precipitated by adding isopropanol (Merck) and centrifuged again. The resulting protein pellet was washed three times with 2 mL wash solution containing 0.3 M guanidine hydrochloride (Merck) in 95% ethanol, followed by centrifugation at 7500×*g* for 5 min. After the third wash, 100% ethanol was added to the pellet, incubated at room temperature for 20 min, and centrifuged at 7500×*g* for 5 min. The protein pellet was air‐dried for 10 min and resuspended in 200 μL of 1% SDS solution. The total protein content was determined using the BCA Protein Assay Kit (Pierce) according to the manufacturer's instructions.

### 4.8 Total and Histone Protein Extraction From SCs

For extraction of total proteins, SCs were collected after enzymatic digestion with trypsin (Euroclone), lysed by sonication in PBS containing 1% protease inhibitor cocktail and 1% phosphatase inhibitor cocktail, and centrifuged at 800×*g* for 10 min.

For extraction of histone proteins, the pellet of SCs was then resuspended in 400 μL lysis buffer (10 mM HEPES pH 7.4, 10 mM KCl, 0.05% Igepal, 1% protease inhibitor cocktail, and 1% phosphatase inhibitor cocktail) and incubated on ice for 20 min. The sample was then centrifuged at 17,000×*g* for 15 min. The pellet was resuspended in 200 μL Low Salt Buffer (LSB, 10 mM Tris–HCl pH 7.4, 0.02 mM MgCl2, 1% Triton X‐100, 1% protease inhibitor cocktail and 1% phosphatase inhibitor cocktail), incubated on ice for 15 min and centrifuged at 17,000×*g* for 10 min to isolate the chromatin (pellet) from the nucleoplasm (supernatant). The chromatin was resuspended in 70 μL HCl 0.2 N (Merck) and incubated on ice for 30 min. The effect of HCl was neutralized by adding the same amount of Tris–HCl pH 8. The chromatin was centrifuged at 17,000×*g* for 10 min to separate the histones (supernatant) from the DNA (pellet). The protein content was determined using the BCA Protein Assay Kit (Thermo Fisher Scientific) according to the manufacturer's instructions.

### 4.9 Western Blot

Proteins were denatured by boiling for 5 min in sample buffer (0.6 g/100 mL Tris, 2 g/100 mL SDS, 10% glycerol, 1% 2‐mercaptoethanol, pH 6.8). The histone proteins were loaded onto a 15% SDS‐PAGE gel, while the total proteins were loaded onto a 10% gel. After electrophoresis, the proteins were transferred to a nitrocellulose membrane (0.45 μm, Bio‐Rad) by electroblotting. The nitrocellulose membranes were blocked for 1 h in 5% (w/v) non‐fat dry milk (Bio‐Rad) in Tris‐buffered saline/0.1% Tween‐20 (TBS‐T) and then incubated overnight (4°C) with rabbit monoclonal HIF‐1α (clone D2U3T, Cell Signaling; 1:1000 in 5% BSA/TBS‐T), rabbit monoclonal HIF‐2α (clone D9E3, Cell Signaling; 1:1000 in 5% non‐fat dry milk/TBS‐T), mouse polyclonal histone H3 (clone D1H2, Cell Signaling; 1:1000 in 5% BSA/TBS‐T) or rabbit monoclonal p16^INK4A^ (clone E6N8P, Cell Signaling; 1:1000 in 5% non‐fat dry milk/TBS‐T); membranes designated for lactylation detection were incubated for 2 h at room temperature with mouse monoclonal Pan KIaH4K5Lac (PTM BIO; 1:1000 in 5% non‐fat dry milk/TBS‐T). After three 5‐min washes in TBS‐T, blots were probed for 1 h with the corresponding HRP‐conjugated secondary antibody—anti‐rabbit (CiteAb, 1:10000) or anti‐mouse (GE Healthcare, 1:3000)—and developed as described.

After washing three times for 5 min with TBS‐T, the chemiluminescence signal on the membrane was developed using Western Supernova detection reagent (Cyanagen), and the resulting signal was recorded using the LI‐COR Odyssey infrared imaging system. The relative band intensities were quantified using LI‐COR Image Studio Light (version 5.2), and the results were normalized to the total amount of transferred proteins detected using the REVERT Total Protein Stain Kit (LI‐COR Biotechnology) according to the manufacturer's instructions.

### 4.10 CellTiter‐GLO

After treatment with 50 μM FG‐4592, 4 × 10^3^ SCs/well were seeded in a 96‐well low‐adherent white luminescent plates. The cellular suspension was incubated with an equal volume of Celltiter‐GLO (Promega) substrate for 10 min to measure the intracellular adenosine triphosphate (ATP) concentration. Then, luminescence was recorded using a PerkinElmer multi‐label plate reader, VICTOR (Thermo Fisher Scientific).

### 4.11 Bioluminescent Assessment of Glucose Uptake and Intracellular Lactate Production

Both bioluminescence assays (Promega) were performed using 4 × 10^3^ SCs/well seeded 24 h before treatment with 50 μM FG‐4592 for 48 h into 96‐well Low Adherent White Luminescent plates.

To measure the glucose uptake, SCs were washed with 100 μL PBS, and 1 mM 2‐Deoxy‐D‐Glucose (2DG) was added to start the assay. After stopping the reaction, the samples were processed as described in the standard Glucose Uptake‐Glo Assay Kit (Promega) protocol. Since glucose uptake is time‐dependent, the optimal time length was determined by stopping the reaction after 1 h. This is the time frame chosen for the standard conditions of glucose uptake in SCs. Luminescence was then recorded using the Perkin Elmer VICTOR multi‐label plate reader. The rate of glucose uptake was calculated by considering the number of cells (counted by trypan blue exclusion), the time of uptake (1 h), and the amount of 2‐Deoxy‐D‐Glucose‐6‐phosphate (2DG6P) (μM) produced.

To measure the intracellular lactate production, SCs were washed with 100 μL PBS, and the inactivation solution was added to stop metabolism, lyse the cells, and inhibit the activity of endogenous proteins that destroy reduced NADP(P)H dinucleotides. The reaction was inhibited by adding the neutralization mixture. Then, lactate was measured by adding Lactate Detection Reagent (containing lactate dehydrogenase, NAD^+^, reductase, reductase substrate, and luciferase) to SCs. The reaction was incubated for 1 h at room temperature. Luminescence was then recorded using the PerkinElmer VICTOR multi‐label plate reader. The rate of intracellular lactate was calculated by considering the number of cells (counted by trypan blue exclusion) and the time of uptake (1 h).

### 4.12 Cell Cycle Analyses

SCs were grown for 24 h in a GM without FBS and HS to synchronize their cell cycle. Then, SCs were treated with 50 μM FG‐4592 for 48 h to be collected (2 × 10^6^ cells/ml), and centrifuged at 500×*g* for 5 min. The pellet was previously resuspended in 25 μL PBS + 2% FBS, and cold 70% ethanol was added dropwise while vortexing SCs to minimize the formation of cell aggregates. The SCs were incubated on ice for 60 min and centrifuged at 500×*g* for 5 min. After washing twice with PBS + 2% FBS, 250 μL Propidium Iodide (PI) solution (Merck), containing PI at a concentration of 0.1 mg/mL in PBS, was added to label SCs, and 250 μL RNase solution (2 mg/mL in PBS) to eliminate the possible presence of RNA. Finally, SCs were incubated in the dark for 30 min before the Cytoflex S flow cytometer reading (Beckman Coulter).

### 4.13 Immunofluorescence

SCs were washed twice with PBS and fixed in 1 mL of 4% (w/v) paraformaldehyde (PFA) in PBS and incubated for 15 min in the dark. After fixation, the PFA was removed by washing the cells three times with PBS.

For PAX7 staining, SCs were permeabilized for 20 min at room temperature with a solution containing 0.3% (w/v) Triton X‐100. The SCs were washed 3 times with PBS and incubated for 1 h with a blocking solution of 3% (w/v) BSA in PBS and then overnight at 4°C with a mouse anti‐Pax7 antibody diluted 1:13 in blocking solution.

For MHC staining, SCs were incubated for 1 h with a blocking solution, consisting of 5% FBS and 0.1% (p/v) Triton X‐100, and then incubated for 2 h with the mouse anti‐MHC antibody (Merck), diluted 1:200 in blocking solution.

For both staining procedures, SCs were washed three times with PBS and incubated with the secondary anti‐mouse antibody (Fitch, Jackson Laboratories) diluted 1:200 in appropriate blocking solution for 1 h at room temperature. Finally, the SCs were incubated with DAPI (4′,6‐diamidino‐2‐phenylindole, Merck) diluted in blocking solution and analyzed with AXIO Observer (Zeiss).

### 4.14 Statistics

All tests were performed with three to six replicates, and quantitative data are expressed as mean ± standard error of the mean (SEM). The nonparametric Mann–Whitney test or one‐way ANOVA and Dunnet test for multiple comparisons were used to determine statistical significance using GraphPad Prism 9 software. *p*‐values of < 0.05 were considered significant.

For liquid chromatography‐mass spectrometry (LC–MS) analysis, raw data were processed using Proteome Discoverer 2.5 software (PD, Thermo Fisher Scientific). All spectra were submitted to the Sequest HT search engine for protein identification using the Musculus database (downloaded from the Uniprot database on March 13, 2022, 42280 sequences) and a common contaminant database from Thermo Fisher Scientific. The identification parameters were: 2 trypsin cleavage sites, precursor mass tolerance of 10 ppm and fragment mass tolerance of 0.02 Da; carbamidomethyl, oxidation, protein N‐terminal acetylation, loss of methionine and Met loss+acetyl were defined as dynamic modifications. The false discovery rate (FDR) was set to 5% as calculated with the Percolator algorithm in the Proteome Discoverer workflow. The Precursor Ions Quantifier node was used for quantification using the following parameters. Peptide: unique; Precursor abundance based on: Intensity; Normalization Mode: total peptide amount; Protein Ratio Calculation: pairwise ratio based; Hypothesis Test: *t*‐test (background based). All other parameters were set as standard. Differentially expressed proteins between analyzed groups were determined considering the following filters: (a) FDR confidence: HIGH; (b) impurities excluded; (c) peak found in at least 80% of samples; (d) *p*‐value set to ≤ 0.05; (e) adjusted *p*‐value ≤ 0.05; (f) log2 fold change was set to 0.3. Volcano plot analysis, PCA, and heatmap were performed with PD software after applying the filters described above.

## Author Contributions

Federica Cirillo, Marco Piccoli, and Luigi Anastasia conceived the study, designed the experiments, and performed the data analysis. They also wrote the manuscript and prepared the figures. Ivana Lavota, Paola Rota, and Lorenzo Mornatti reviewed and revised the manuscript. Federica Cirillo and Marco Piccoli were responsible for killing the mice and harvesting the skeletal muscle tissue, while Lorenzo Mornatti and Monica Risuglia isolated the SCs. Pasquale Creo and Adriana Tarantino performed the cytofluorimeter analysis, while Ivana Lavota and Paola Rota performed the LC–MS study. Elena Vizzino performed the revisions. Lorenzo Mornatti, Giuseppe Maria Peretti, Paola Signorelli, Simone Cenci, and Luigi Anastasia provided financial support for the project. Giuseppe Ciconte and Carlo Pappone supervised the design and execution of the research. All authors reviewed the results and approved the final version of the manuscript.

## Funding

This work wassupported by the Ministry of Health. Ricerca Corrente to IRCCS Policlinico San Donato and Next generation EU, DM 1557 11.10.2022.

## Conflicts of Interest

The authors declare no conflicts of interest.

## Supporting information


**Figure S1:** Characterization of HIF‐1α and HIF‐2α antibody. C2C12 cells treated with DMSO or FG‐4592 were used to assess antibody specificity for HIF‐1α and HIF‐2α. Following FG‐4592 treatment, a specific band at ~120 kDa was detected for both proteins.

**Figure S2:** Satellite cells characterization phenotype. (a) Flow cytometry gating of muscle‐derived cells to isolate satellite cells (SCs). The red represents unstained cells (negative control), while the green represents the stained population. Panels show sorted cells stained for: CD106‐positive SCs (i panel), CD11‐positive cells (granulocytes, monocytes, and macrophages, ii panel), CD31‐positive cells (endothelial cells, iii panel), CD45‐positive cells (leukocytes, iv panel), and CD34‐positive cells (hematopoietic stem cells, v panel). Gene expression of: (i) *Pax7* in SCs, Skeletal muscle fibroblasts, adipocytes, and endothelial cells, (ii) *Thy1, Pdgfra*, *Fabp4* in skeletal muscle fibroblasts and SCs, (iii) *Pparg* and *Plin1* in adipocytes and SCs, (iv) *Pecam‐1* and *Vwf* in endothelial cells and SCs.

**Figure S3:** Proteomic differences between young and old DMSO‐control SCs. (a) Volcano plot of −log_10_ *p*‐value versus log_2_ change and graphs showing the significantly different abundance of proteins in young and old untreated SCs. (b) Heatmap of proteins modulated in SCs during aging and characterized by a *p*‐value ≤ 0.05 and a log_2_FC ≥ 0.3. The color in each tile represents the scaled abundance value. (c, d) GO analysis of functional enrichment and pathway database (KEGG, Reactome, WikiPathways) by SRPlot in young and old untreated SCs.

**Figure S4:** Molecular and proteomic response to FG‐4592 treatment in young and old SCs. (a) Evaluation of *Vegfa* and *Egln1* gene expression by Real‐Time PCR in young and old SCs treated with FG‐4592 as compared to DMSO‐controls. (b) Volcano plot of −log_10_ *p*‐value versus log_2_ showing the significantly different abundance of proteins induced by FG‐4592 treatment compared to DMSO‐control in young and old SCs. (c) Heatmap of proteins modulated by the treatment and characterized by a *p*‐value ≤ 0.05 and a log_2_FC ≥ 0.6. The color in each tile represents the scaled abundance value. Data represent mean ± SEM. Statistical significance was determined by one‐way ANOVA. *p < 0.05, **p < 0.01, ****p < 0.0001.

**Figure S5:** Network and functional enrichment analysis in young and old SCs co‐treated with oxamate and FG‐4592. (a) Volcano plot of −log_10_ *p*‐value versus log_2_ showing the significantly different abundance of proteins induced by the co‐treatment compared to DMSO‐control in young and old SCs. (b) Heatmap of proteins modulated by the treatment and characterized by a *p*‐value ≤ 0.05 and a log_2_FC ≥ 0.6. The color in each tile represents the scaled abundance value. (c) GO analysis of functional enrichment and pathway database (KEGG, Reactome, Wikipathways) by SRPlot in young and old co‐treated SCs as compared to the DMSO‐controls.

**Figure S6:** Network and functional enrichment analysis in young and old SCs myotubes. (a) Protein–protein interaction network modulated in young and old DMSO‐control myotubes. (c) GO analysis of functional enrichment and pathway database (KEGG, Reactome, WikiPathways) by SRPlot in young and old DMSO‐control myotubes.

**Figure S7:** Differential protein abundance and expression patterns in FG‐4592–treated young and old myotubes. (a) Volcano plot of −log_10_ *p*‐value versus log_2_ change showing the significantly different abundance of proteins induced by FG‐4592 treatment in young and old myotubes as compared to DMSO‐control myotubes. (b) Heatmap of proteins modulated by the treatment during differentiation and characterized by a *p*‐value ≤ 0.05 and a log_2_FC ≥ 0.3. The color in each tile represents the scaled abundance value. (c, e) Protein–protein interaction network modulated in young (c) and old (e) myotubes generated following FG‐4592 treatment. (d, f) GO analysis of functional enrichment and pathway database (KEGG, Reactome, WikiPathways) by SRPlot in young (d) and old (f) myotubes.

**Figure S8:** Network and functional enrichment analysis in young and old derived myotubes co‐treated with oxamate and FG‐4592. (a) Volcano plot of −log_10_ *p*‐value versus log_2_ showing the significantly different abundance of proteins induced by the co‐treatment compared to DMSO‐control in young and old derived myotubes. (b) Heatmap of proteins modulated by the treatment and characterized by a *p*‐value ≤ 0.05 and a log_2_FC ≥ 0.6. The color in each tile represents the scaled abundance value. (c) GO analysis of functional enrichment and pathway database (KEGG, Reactome, Wikipathways) by SRPlot in young and old co‐treated derived myotubes as compared to the DMSO‐controls.

**Table S1:** List of proteins identified in pathway enrichment analyses. Comparisons between (a) young vs. old (SCs) treated with DMSO, (b) young, and (c) old SCs treated with FG‐4592 vs. DMSO, (d) young and (e) old SCs co‐treated with oxamate and FG‐4592 vs. DMSO.

**Table S2:** List of proteins identified in pathway enrichment analyses. Comparisons between (a) young and (b) old myotubes derived from SCs treated with FG‐4592 vs. DMSO, (c) young and (d) old myotubes derived from SCs co‐treated with oxamate and FG‐4592 vs. DMSO.

**Table S3:** List of primers used for gene expression analysis.

## Data Availability

The data that support the findings of this study are available on request from the corresponding author.
