# Supplementary figures and images for: The HIF‐1α Pathway Regulates Satellite Cell Fate During Aging Through Histone Lactylation
Source: Aging Cell. 2026 Feb 13;25(2):e70411. doi: 10.1111/acel.70411 (PMC12904840; doi:10.1111/acel.70411)

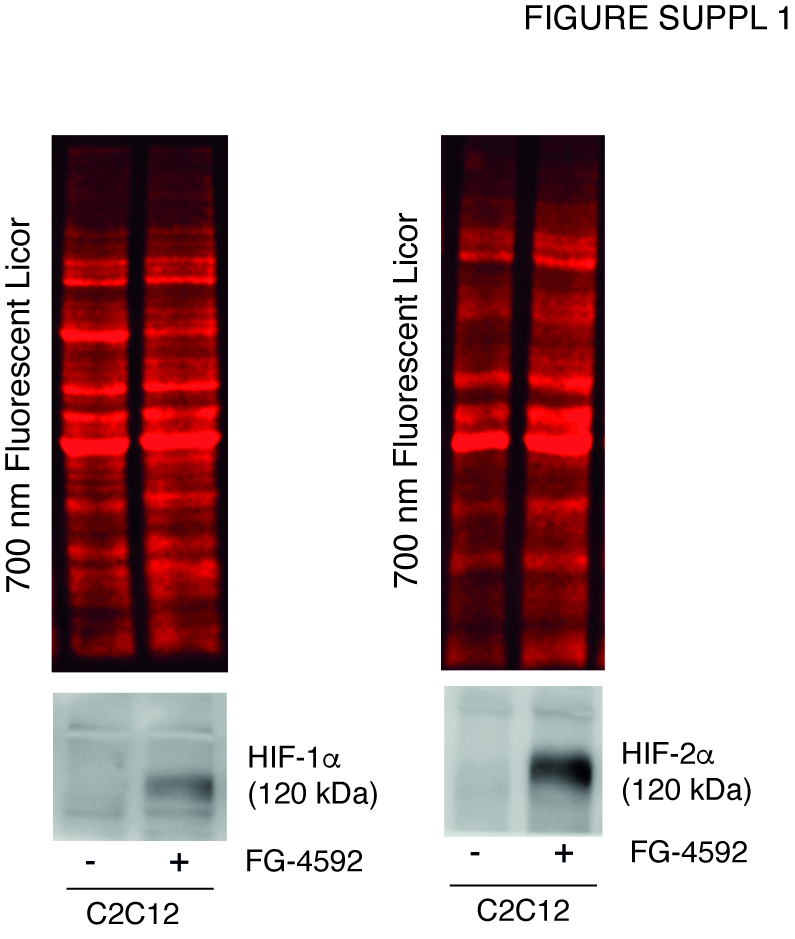

Supplement: Supplementary file 1 — Figure S1: Characterization of HIF‐1α and HIF‐2α antibody. C2C12 cells treated with DMSO or FG‐4592 were used to assess antibody specificity for HIF‐1α and HIF‐2α. Following FG‐4592 treatment, a specific band at ~120 kDa was detected for both proteins. [file ACEL-25-e70411-s005.tif]

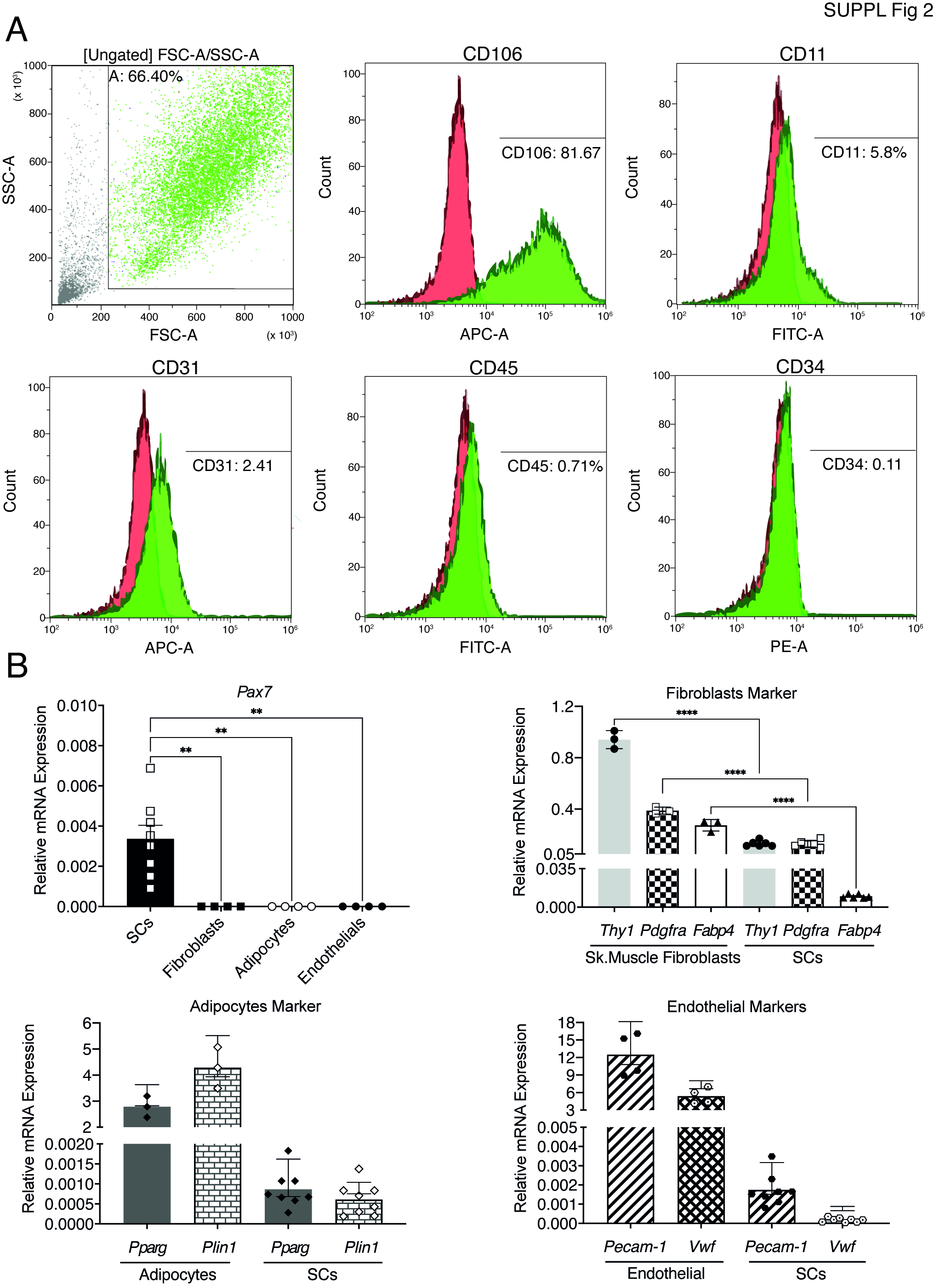

Supplement: Supplementary file 2 — Figure S2: Satellite cells characterization phenotype. (a) Flow cytometry gating of muscle‐derived cells to isolate satellite cells (SCs). The red represents unstained cells (negative control), while the green represents the stained population. Panels show sorted cells stained for: CD106‐positive SCs (i panel), CD11‐positive cells (granulocytes, monocytes, and macrophages, ii panel), CD31‐positive cells (endothelial cells, iii panel), CD45‐positive cells (leukocytes, iv panel), and CD34‐positive cells (hematopoietic stem cells, v panel). Gene expression of: (i) Pax7 in SCs, Skeletal muscle fibroblasts, adipocytes, and endothelial cells, (ii) Thy1, Pdgfra, Fabp4 in skeletal muscle fibroblasts and SCs, (iii) Pparg and Plin1 in adipocytes and SCs, (iv) Pecam‐1 and Vwf in endothelial cells and SCs. [file ACEL-25-e70411-s006.tif]

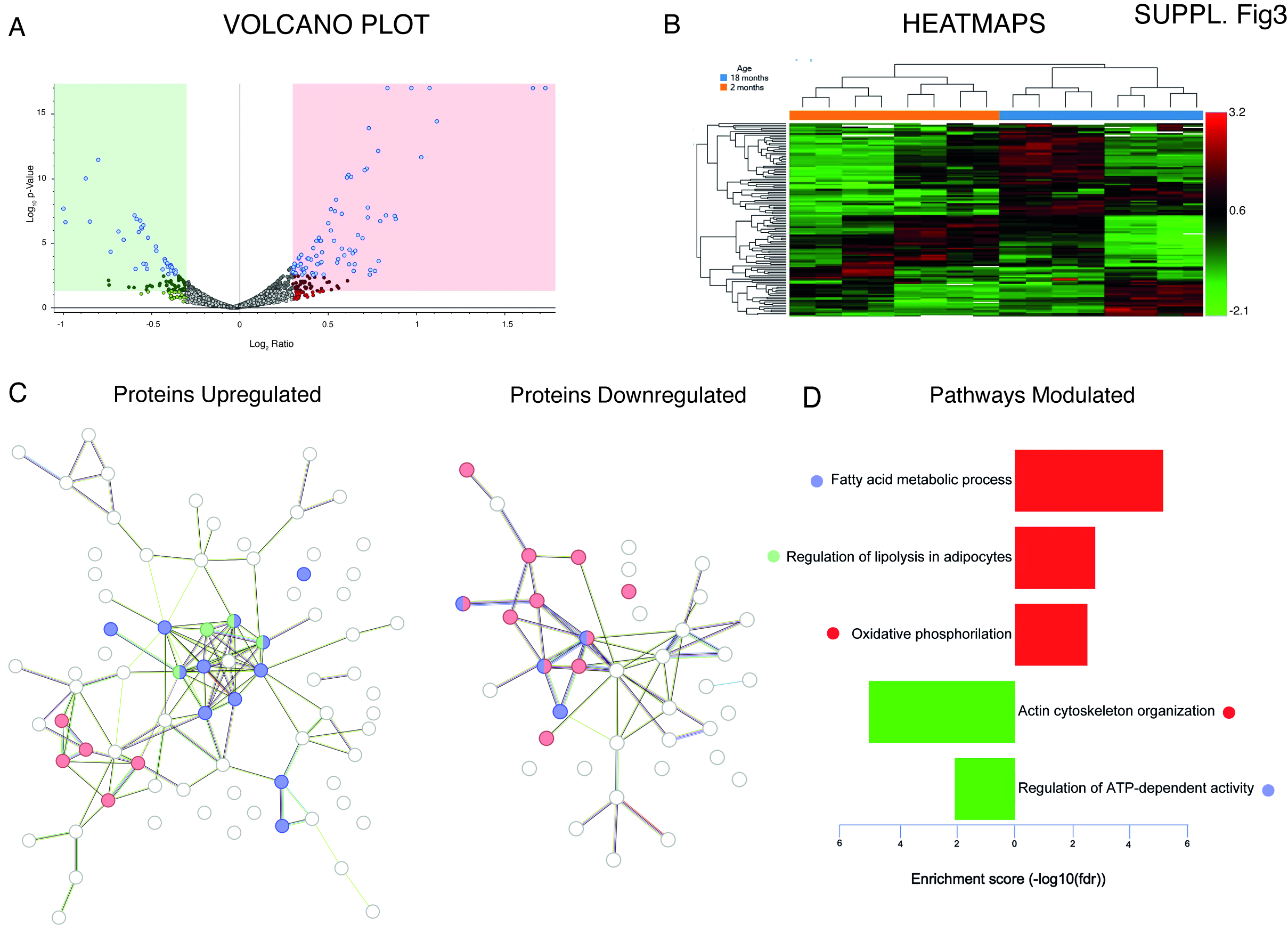

Supplement: Supplementary file 3 — Figure S3: Proteomic differences between young and old DMSO‐control SCs. (a) Volcano plot of −log10 p‐value versus log2 change and graphs showing the significantly different abundance of proteins in young and old untreated SCs. (b) Heatmap of proteins modulated in SCs during aging and characterized by a p‐value ≤ 0.05 and a log2FC ≥ 0.3. The color in each tile represents the scaled abundance value. (c, d) GO analysis of functional enrichment and pathway database (KEGG, Reactome, WikiPathways) by SRPlot in young and old untreated SCs. [file ACEL-25-e70411-s009.tif]

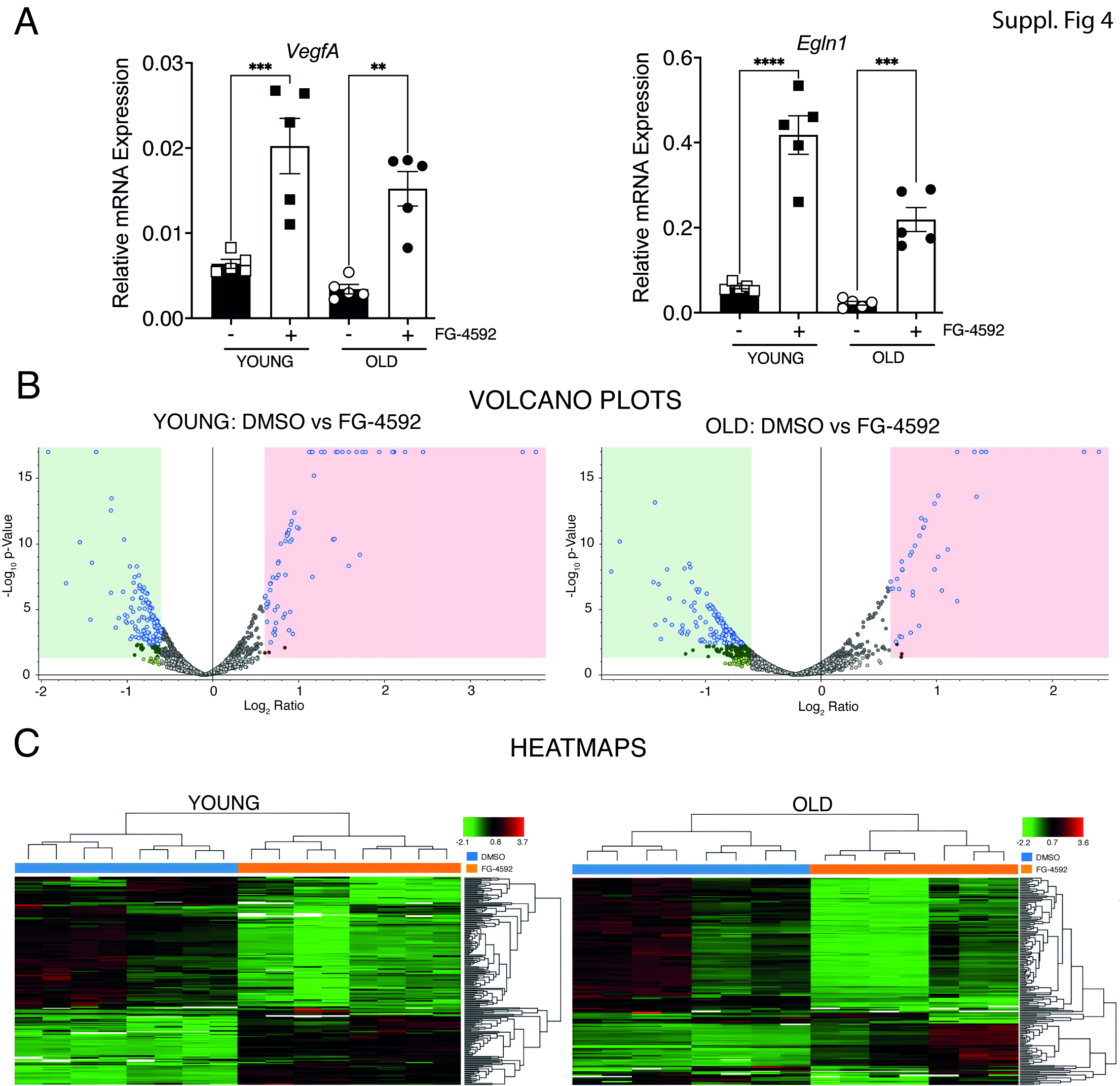

Supplement: Supplementary file 4 — Figure S4: Molecular and proteomic response to FG‐4592 treatment in young and old SCs. (a) Evaluation of Vegfa and Egln1 gene expression by Real‐Time PCR in young and old SCs treated with FG‐4592 as compared to DMSO‐controls. (b) Volcano plot of −log10 p‐value versus log2 showing the significantly different abundance of proteins induced by FG‐4592 treatment compared to DMSO‐control in young and old SCs. (c) Heatmap of proteins modulated by the treatment and characterized by a p‐value ≤ 0.05 and a log2FC ≥ 0.6. The color in each tile represents the scaled abundance value. Data represent mean ± SEM. Statistical significance was determined by one‐way ANOVA. *p < 0.05, **p < 0.01, ****p < 0.0001. [file ACEL-25-e70411-s007.tif]

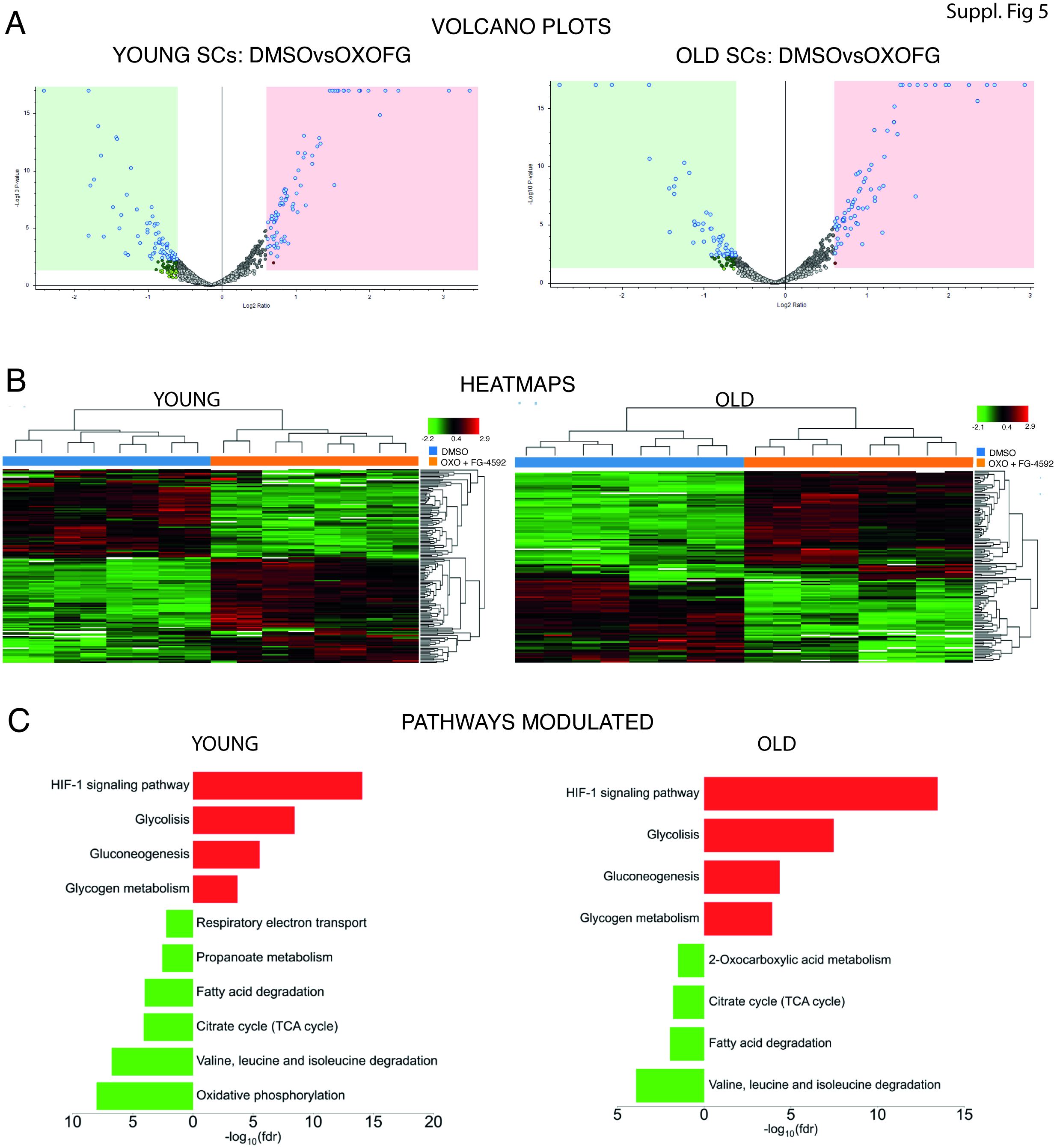

Supplement: Supplementary file 5 — Figure S5: Network and functional enrichment analysis in young and old SCs co‐treated with oxamate and FG‐4592. (a) Volcano plot of −log10 p‐value versus log2 showing the significantly different abundance of proteins induced by the co‐treatment compared to DMSO‐control in young and old SCs. (b) Heatmap of proteins modulated by the treatment and characterized by a p‐value ≤ 0.05 and a log2FC ≥ 0.6. The color in each tile represents the scaled abundance value. (c) GO analysis of functional enrichment and pathway database (KEGG, Reactome, Wikipathways) by SRPlot in young and old co‐treated SCs as compared to the DMSO‐controls. [file ACEL-25-e70411-s004.tif]

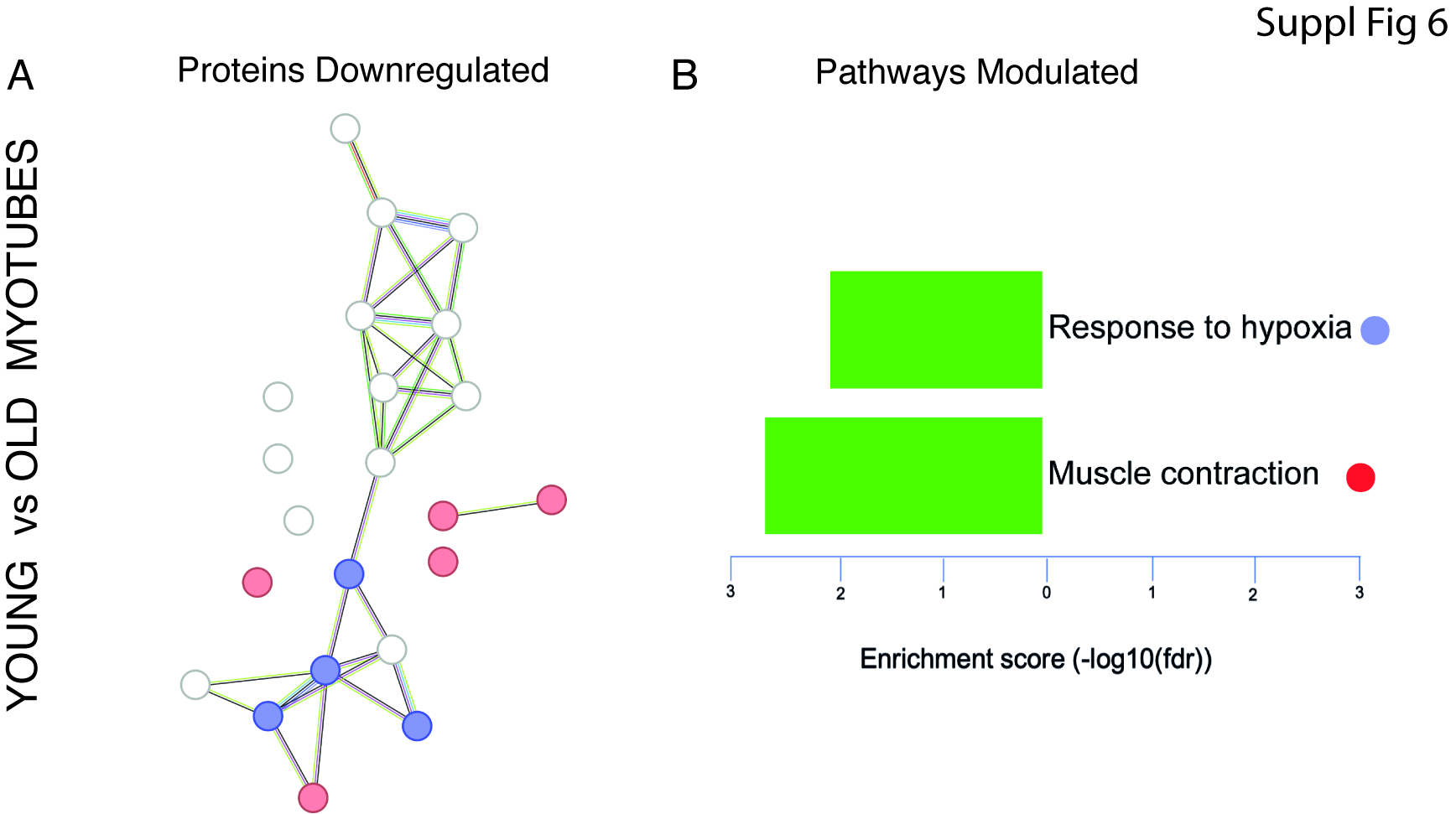

Supplement: Supplementary file 6 — Figure S6: Network and functional enrichment analysis in young and old SCs myotubes. (a) Protein–protein interaction network modulated in young and old DMSO‐control myotubes. (c) GO analysis of functional enrichment and pathway database (KEGG, Reactome, WikiPathways) by SRPlot in young and old DMSO‐control myotubes. [file ACEL-25-e70411-s003.tif]

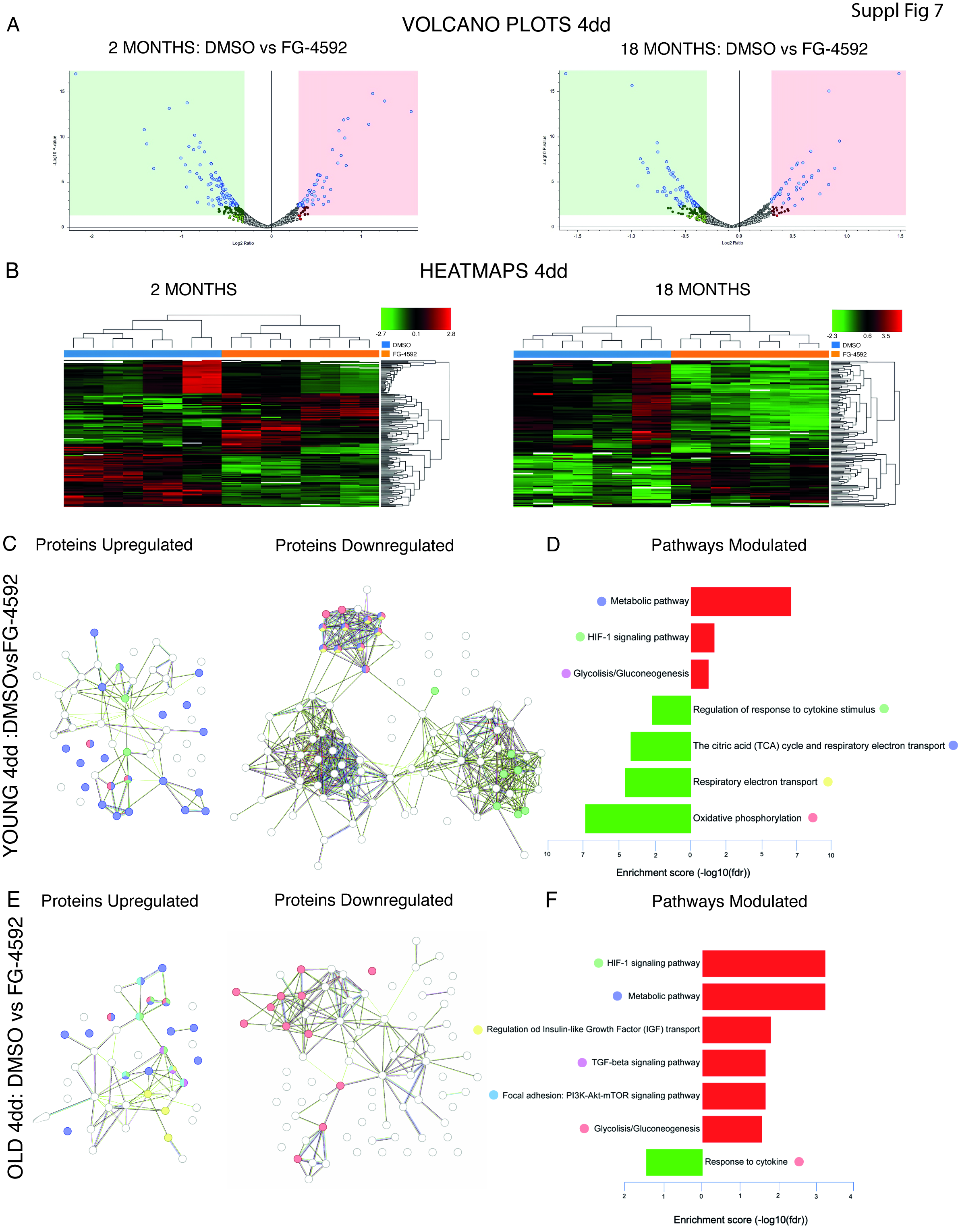

Supplement: Supplementary file 7 — Figure S7: Differential protein abundance and expression patterns in FG‐4592–treated young and old myotubes. (a) Volcano plot of −log10 p‐value versus log2 change showing the significantly different abundance of proteins induced by FG‐4592 treatment in young and old myotubes as compared to DMSO‐control myotubes. (b) Heatmap of proteins modulated by the treatment during differentiation and characterized by a p‐value ≤ 0.05 and a log2FC ≥ 0.3. The color in each tile represents the scaled abundance value. (c, e) Protein–protein interaction network modulated in young (c) and old (e) myotubes generated following FG‐4592 treatment. (d, f) GO analysis of functional enrichment and pathway database (KEGG, Reactome, WikiPathways) by SRPlot in young (d) and old (f) myotubes. [file ACEL-25-e70411-s008.tif]

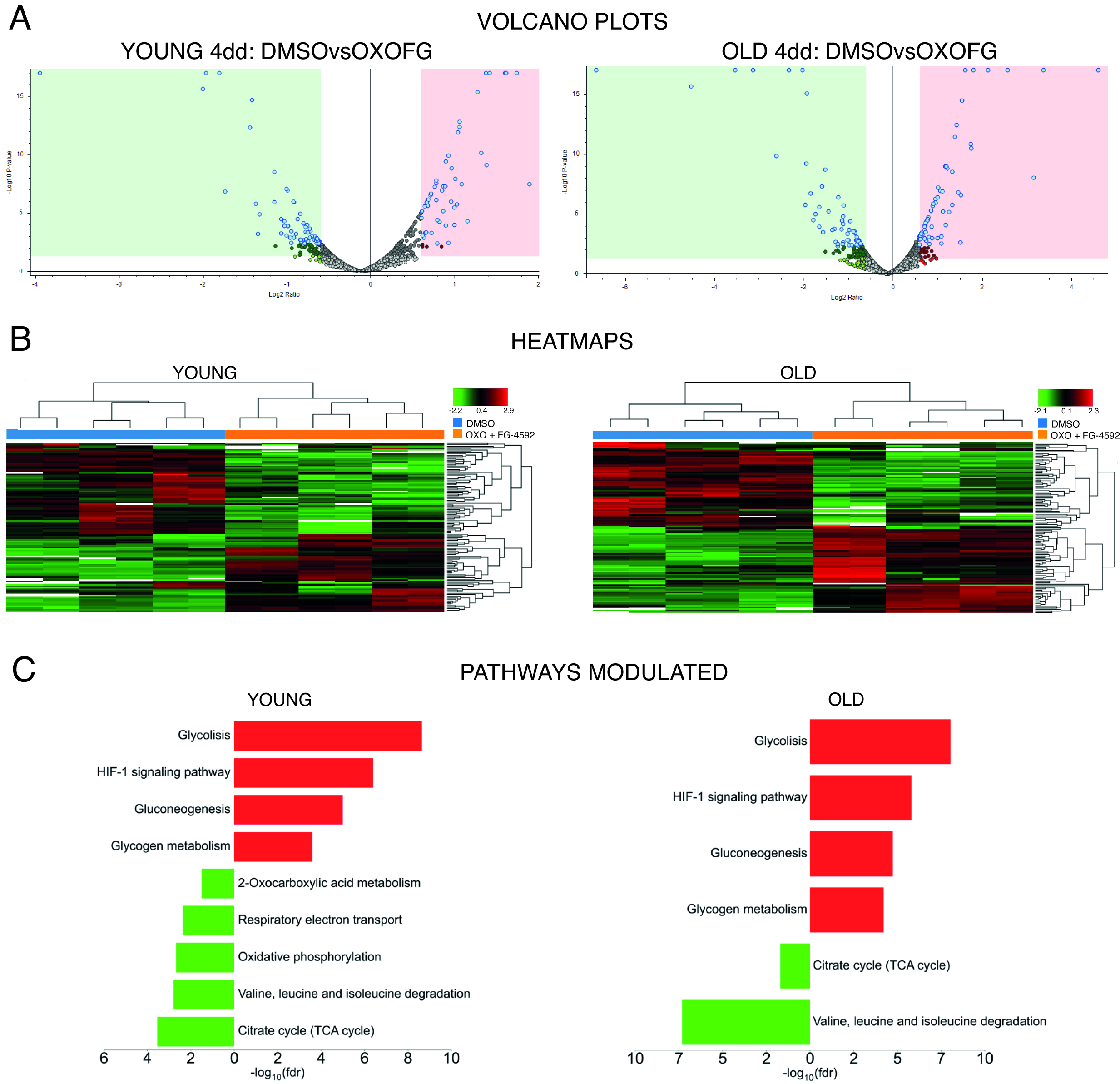

Supplement: Supplementary file 8 — Figure S8: Network and functional enrichment analysis in young and old derived myotubes co‐treated with oxamate and FG‐4592. (a) Volcano plot of −log10 p‐value versus log2 showing the significantly different abundance of proteins induced by the co‐treatment compared to DMSO‐control in young and old derived myotubes. (b) Heatmap of proteins modulated by the treatment and characterized by a p‐value ≤ 0.05 and a log2FC ≥ 0.6. The color in each tile represents the scaled abundance value. (c) GO analysis of functional enrichment and pathway database (KEGG, Reactome, Wikipathways) by SRPlot in young and old co‐treated derived myotubes as compared to the DMSO‐controls. [file ACEL-25-e70411-s010.tif]
