# Supplementary material for: The HIF‐1α Pathway Regulates Satellite Cell Fate During Aging Through Histone Lactylation
Source: Aging Cell. 2026 Feb 13;25(2):e70411. doi: 10.1111/acel.70411 (PMC12904840; doi:10.1111/acel.70411)
Supplement: Supplementary file 11 — Table S3: List of primers used for gene expression analysis. [file ACEL-25-e70411-s002.docx]

Supplementary Table 1

| PRIMERS | SEQUENCE |
| --- | --- |
| *Rpli* | FW: 5’ CTC GGC CGT TCT CCT GTA T 3’ RW: 5’ GTG GAA GTG GGG CTT CAG TA 3’ |
| *Vegfa* | FW: 5’ AAA ACC TCA CCA AAG CCA G 3’  RW: 5’ ACA GTG AAC GCT CCA GGA T 3’ |
| *Egln1* | FW: 5’ CTG TGG AAC AGC CCT TTT TG 3’  RW: 5’ CGA CTC TCT CTG CGA ATC C 3’ |
| *Aldo* | FW: 5’ GTG GGA AGA AGG AGA ACC TG 3’  RW: 5’ CTG GAG TGT TGA TGG AGC AG 3’ |
| *Gapdh* | FW: 5’ CGT GCC GCC TGG AGA AAC 3’  RW: 5’ TGG GAG TTG CTG TTG AAG TCG 3’ |
| *Aco2* | FW: 5’ GTC GCC ACC ATT CTT TGA A 3’  RW: 5’ GGC ATC CAC TAT GGA CTT GG 3’ |
| *Pdk2* | FW: 5’ AAA GAG ATC AAC CTG CTT CC 3’  RW: 5’ TTC CAT GAT GTC CAG CAG AC 3’ |
| *Pdha1* | FW:5’GCA TCG TTG GAG CTC AG 3’ RW:5’ACA GAC CTC ATC TTT TCC ATT GT 3’ |
| *Ldha* | FW:5’ TCCGTTACCTGATGGGAGAG 3’ RW:5’ GCAACATTCACACCACTCCA 3’ |
| *Cdk1* | FW: 5’ CGGCGAGTTCTTCACAGAGA 3’ RW: 5’ CTGGAGATCGGTACCACAG 3’ |
| *Cdk2* | FW: 5’ TCTGCCATTCTCACCGTGTC 3’ RW: 5’ GACAGGGACTCCAAAGGCT 3’ |
| *Ccna2* | FW: 5’ CCCAGTACTTCCTGCACCT 3’ RW: 5’ GACCTCTGTCCTGTGACTG 3’ |
| *Ccne1* | FW: 5’ CTCCCACAACATCCAGACC 3’ RW: 5’ TGCTTCTTACTGCTGGGTG 3’ |
| *Slc16a1* | FW: 5’ CAT TGG TGT TAT TGG AGG TC 3’  RW: 5’ GAA AGC GTG ATT AAG TGG AG 3’ |
| *Slc16a3* | FW: 5’ TCA ATC ATG GTG CTYG GGA CT 3’  RW: 5’ TGT CAG GTC AGT GAA GCC AT 3’ |
| *Pax7* | FW: 5’ GGC ACA GAG GAC CAA GC 3’  RW: 5’ GCA CGC CGG TTA CTG AAC 3’ |
| *Tpm1* | FW: 5’ GCA AAT GTG CCG AGC TTG AA 3’  RW: 5’ CAG CCT CCT TCA GCT TGT C 3’ |
| *Tpm2* | FW: 5’ CAA AAG CTG GAG GAG GCT GA 3’  RW: 5’ TTC CTG GCC ACC TCC TCA TA 3’ |
| *Igf-1* | FW: 5’ GAC CGA GGG GCT TTT ACT TC 3’  RW: 5’ CAC TCA TCC ACA ATH CCT GT 3’ |
| *Murf-1* | FW: 5’ TGA CAT CTA CAA GCA GGA GTG C 3’  RW: 5’ TCG TCT TCG TGT TCC TTG C 3’ |
| *Cdkn2a* | FW: 5’ AGG ACC CCA CTA CCT TCT C 3’  RW: 5’ CAG CGG AAC ACA AAG AGC AC 3’ |
| *Thy1* | FW: 5’ TGC CGC CAT GAG AAT AAC AC 3’  RW: 5’ AAG TAG TCG CCC TCA TCC TT 3’ |
| *Pdgfra* | FW: 5’ TAT GAA TGT GCT GCC CGT CA 3’  RW: 5’ AAG TTG TCC TTC AGC CAC GA 3’ |
| *Pecam-1* | FW: 5’ TCG GCA AAG TGG TCA AGA GA 3’  RW: 5’ TGG GTG CAG TTC CAT TTT CG 3’ |
| PERILIPIN | FW: 5’ CAG TTC ACA GCT GCC AAT GA 3’  RW: 5’ TGT CAG AGG TGC TTG CAA TG 3’ |
| *Fabp4* | FW: 5’ AAG AAG TGG GAG TGG GCT TT 3’  RW: 5’ TTT CAT CGA ATT CCA CGC CC 3’ |
| *Vwf* | FW: 5’ GTG TGT CAG CAG CAA TGT GT 3’  RW: 5’ TTG CTG CAG ATC CAT AGG CT 3’ |
| *Pparg* | FW: 5’ ATG GTG CCT TCG CTG ATG C 3’  RW: 5’ AAC TGT GGT AAA GGG CTT GAT GTC 3’ |
